# Polymeric Nanofiltration Membranes with Enhanced Hydrophilic, Morphological, Transport, and Antifouling Properties—A Review

**DOI:** 10.3390/polym18172066

**Published:** 2026-08-25

**Authors:** Mohammad Ebrahimi

**Affiliations:** Institute CARMeN UMR 6064, ENSICAEN, University of Caen Normandy, 6 Bd. du Maréchal Juin, 14050 Caen, France; mohammad.ebrahimi@ensicaen.fr

**Keywords:** nanofiltration, modification methods, polymer blending, nanoparticle incorporation, surface coating, plasma treatment, layer-by-layer assembly, chemical grafting and crosslinking, interfacial polymerization

## Abstract

Nanofiltration membranes have emerged as a crucial class of pressure-driven separation materials, positioned between ultrafiltration and reverse osmosis in terms of selectivity, permeance, operating pressure, and energy consumption. Their ability to remove fine contaminants—including multivalent ions, organic micropollutants, dyes, and macromolecules—has made them essential in water and wastewater treatment, pharmaceutical processing, and various industrial applications. In spite of their growing relevance, the performance of polymeric nanofiltration membranes, such as polyamide, polysulfone, polyethersulfone, polyvinylidene fluoride, and polyimide, is still constrained by weak hydrophilicity and a strong susceptibility to fouling, which collectively decrease permeance, increase operational costs, and shorten membrane lifespan. In recent years, substantial research efforts have focused on designing and engineering the surface chemistry and structural characteristics of nanofiltration membranes to improve water permeance, reduce foulant adhesion, and improve long-term stability. This review provides a comprehensive and comparative assessment of the most recent modification techniques applied to polymer-based nanofiltration membranes. Strategies such as polymer blending, nanoparticle incorporation, physical surface coating, plasma treatment, chemical attachment, layer-by-layer assembly, and interfacial polymerization are critically examined with respect to their effectiveness and practical limitations supported by recent research examples. Special attention is given to how these modification methods affect membrane morphology, hydrophilicity, permeance, and antifouling properties. Eventually, the review highlights emerging ideas and forward-looking design directions that may guide the next generation of nanofiltration membranes toward higher efficiency, improved durability, and broader industrial applicability.

## 1. Introduction

Today, contamination and limited water resources have become serious global concerns, which negatively affect both ecosystems and human life [1,2,3,4]. For hundreds of years, humans have overused and polluted most water supplies, where finding drinkable water has become more challenging than in the past [5,6,7]. A substantial amount of the world’s freshwater supplies is consumed by agricultural activities and industrial processes [8,9,10]. In addition, for decades, wastewater produced by industrial, agricultural, and urban sources—contaminated with toxic and hazardous substances—has not been sufficiently treated [11,12,13]. These circumstances negatively affect quality of water, disturb aquatic ecosystems, and increase the risk of serious diseases like cancer, kidney failure, nervous system disorders, cholera, typhoid, and dysentery [4,5,8,11]. This situation makes it essential to develop efficient and reliable water treatment and purification techniques [14,15,16,17]. Within the range of available water-treatment strategies, membrane technology stands out for its high efficiency and broad applicability [18,19,20].

There has been a considerable rise in the application of pressure-driven membranes—microfiltration (MF), ultrafiltration (UF), nanofiltration (NF), and reverse osmosis (RO)—for water treatment due to their superior separation and transport capabilities [21,22,23,24]. Particularly, NF membranes have long served a wide range of industries, including water and wastewater treatment, food and beverage, pharmaceutical and biotechnology, chemical, textile, oil and gas, power generation, mining and metallurgy, pulp and paper, and environmental recycling industries [25,26,27,28,29,30]. These membranes are typically utilized to eliminate very small particles, such as divalent and multivalent ions (i.e., calcium, magnesium, sulfates), organic molecules, dyes, hardness, pesticides, and other dissolved contaminants [31,32,33,34]. However, NF membranes are not the best choice for separating monovalent ions, such as sodium and chloride [35,36,37]. NF membranes are widely recognized for their moderate permeate flux and high selectivity. This performance is mainly attributed to their smaller pore size range (between 0.001 and 0.01 μm), which is notably lower than that of MF and UF membranes [21,22,38,39,40,41]. Despite the fact that NF membranes are less effective than RO membranes at removing monovalent ions, they are more economical, consume less energy, and operate under lower pressure [42,43,44,45]. Table 1 provides a summary of the key characteristics of MF, UF, NF, and RO membranes relevant to particle removal, including pore size range, operating pressure, energy consumption, and flux classification.

**Figure 1:**
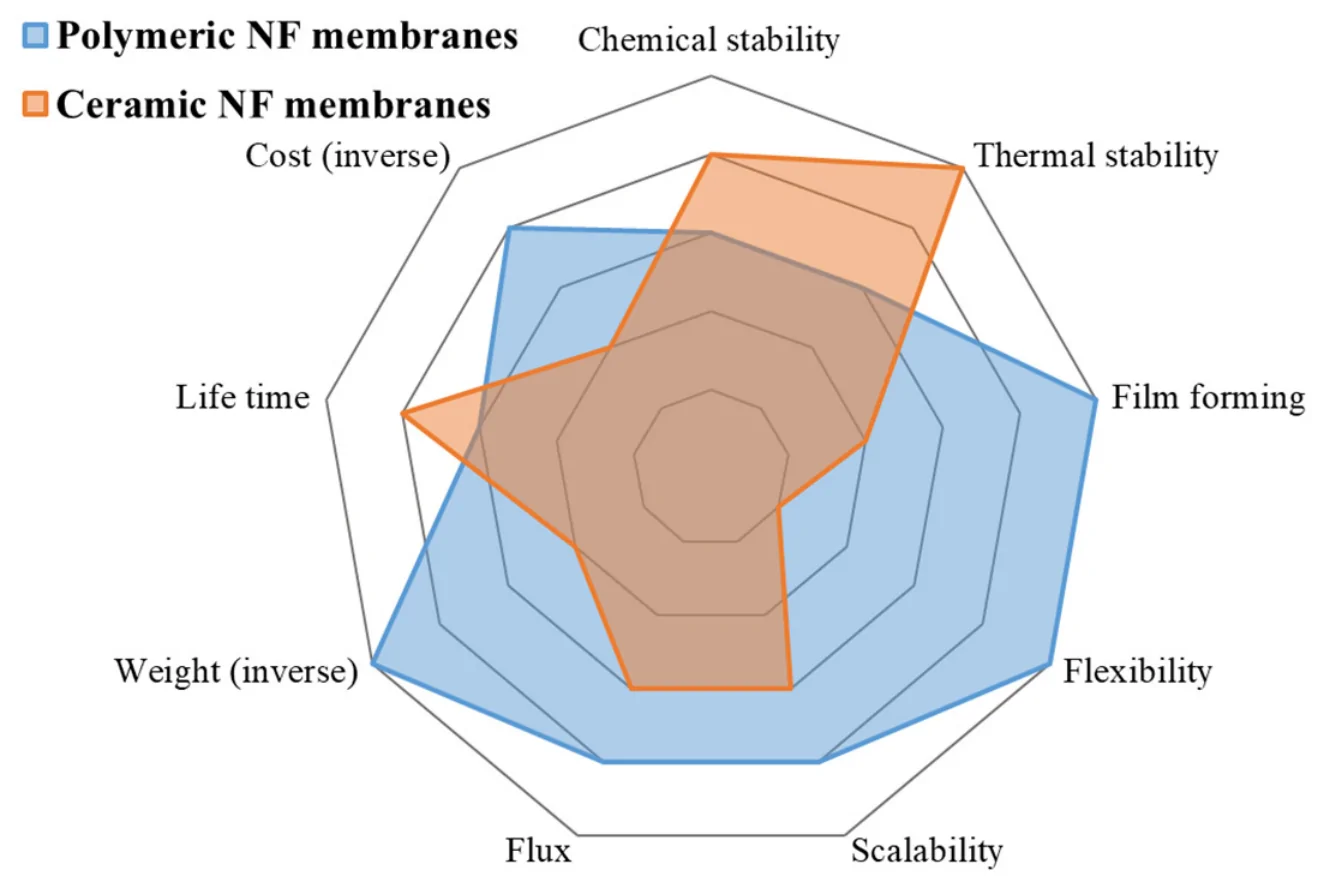
Comparison of polymeric and ceramic NF membranes.

**Figure 2:**
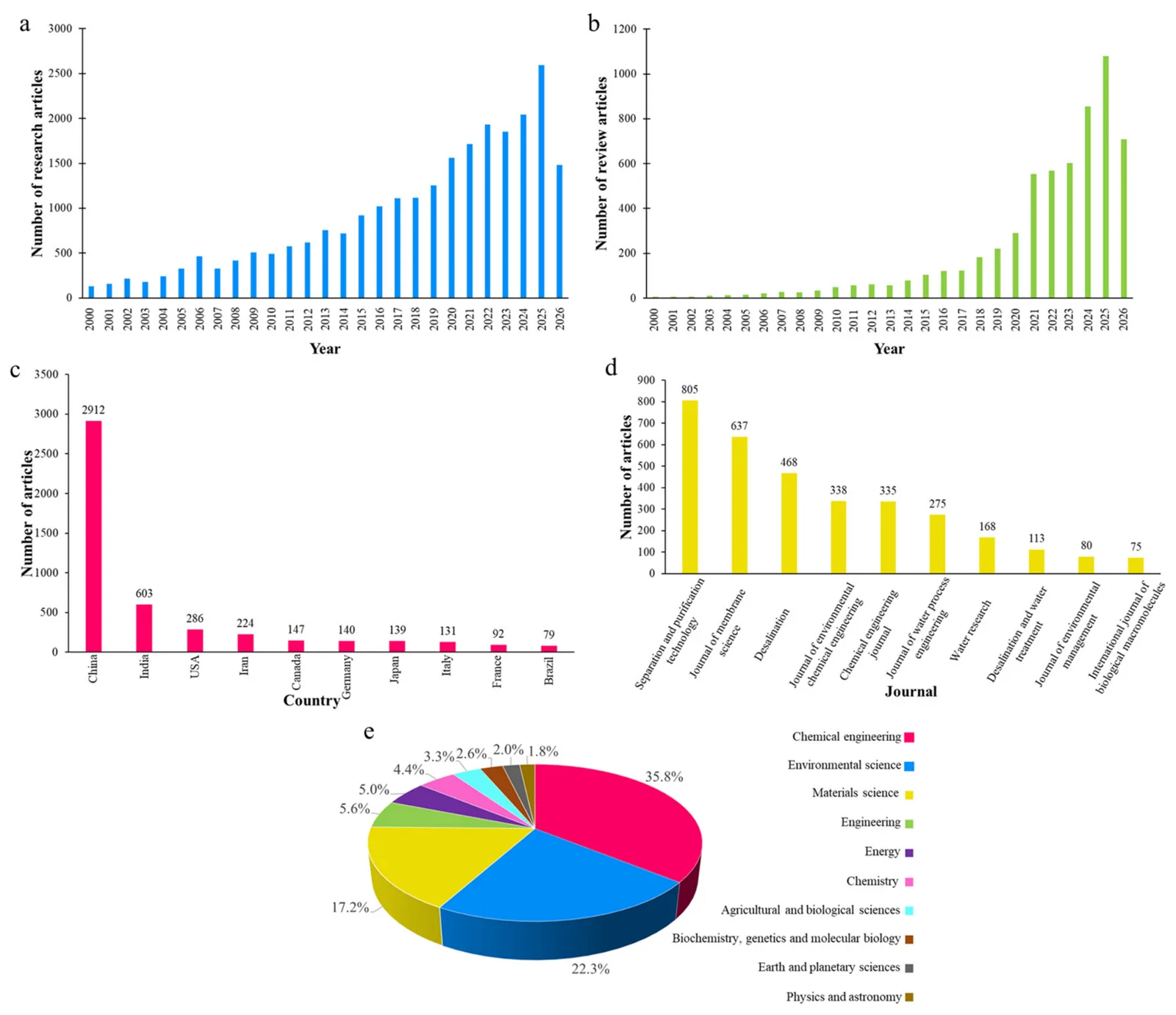
Bibliometric overview of research activity on NF membranes based on Scopus data (June 2026): (**a**) annual number of research articles; (**b**) annual number of review articles; (**c**) top ten contributing countries (2025–2026); (**d**) top ten journals publishing NF-related work (2025–2026); and (**e**) distribution of publication types across the ten most relevant subject areas (2015–2026).

NF membranes are generally classified into two categories—polymeric and ceramic—according to their fabrication materials, and each type possesses its own set of distinct properties [46,47,48,49]. Polyamide (PA), polysulfone (PSf), polyethersulfone (PES), cellulose acetate (CA), polyvinylidene fluoride (PVDF), polyacrylonitrile (PAN), and polyimide (PI) are among the most widely utilized polymers in NF membrane fabrication [50,51,52,53,54,55]. However, ceramic NF membranes are usually made from inorganic materials, including alumina, titania, zirconia, and silica, as well as emerging materials, such as mixed metal oxides, zeolites, and metal–organic frameworks (MOFs) [56,57,58]. Although ceramic NF membranes are known for their enhanced thermal and chemical durability and longer service life, polymeric NF membranes remain advantageous due to their superior film-forming properties, flexibility, scalability potential, and cost-efficiency (Figure 1) [59,60,61]. As a result, polymeric NF membranes have become the preferred option and are widely used in both scientific and industrial sectors [62,63]. Figure 2 provides a brief bibliometric overview of NF membrane research. Figure 2a,b show a steady increase in both research and review publications from 2000 to 2026, reflecting the growing scientific interest in NF membrane development and modification. Figure 2c,d highlight the main contributing countries and journals in recent years, indicating the global and high-impact nature of this research field. Figure 2e summarizes the distribution of publication types across major subject areas, demonstrating the multidisciplinary scope of NF membrane studies.

**Figure 3:**
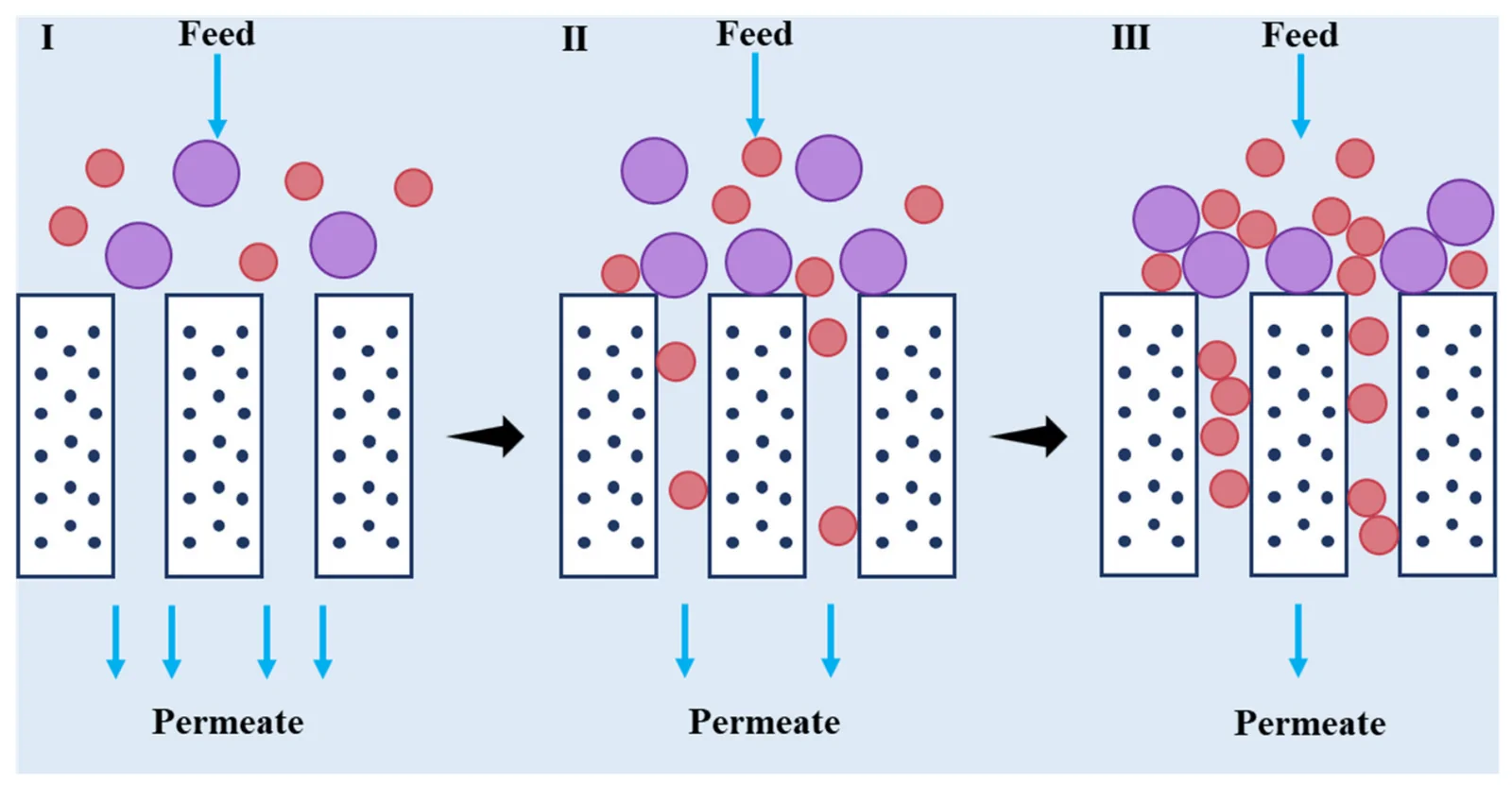
A depiction of the stages of fouling in NF membranes: from early foulant deposition (**I**), to intermediate partial cake layer formation (**II**), and finally to a fully developed cake layer (**III**).

Even though polymeric NF membranes offer several outstanding advantages, these membranes are prone to fouling since the polymers typically used—PA, PSf, CA, PES, PVDF, PAN, and PI—possess inherently insufficient hydrophilic properties [62,64,65]. Membrane fouling is initiated when the membrane surface is hydrophobic, which makes it attractive to contaminants [64,65,66,67]. Therefore, contaminants accumulate, forming a cake layer on the membrane surface, leading to reduced permeate flux, higher energy demand, and a shorter operational service life (Figure 3) [66,68]. Fouling in NF membranes generally develops through various mechanisms, such as pore blockage, cake layer formation, adsorption, and biofouling [69,70]. While the membrane is in use, the surface is exposed to multiple types of contaminants, including organic matter, inorganic salts, colloids, microorganisms, and suspended particles [70,71]. Therefore, based on the nature of these interactions, membrane fouling may occur in two forms: reversible or irreversible [71,72]. Reversible fouling typically occurs when weakly bound foulants—such as colloids, organic matter, and biofilms—accumulate on the membrane surface [39,72,73]. This form of fouling is removable using physical cleaning strategies, such as rinsing with water, backwashing, or sonication [74,75]. In contrast, irreversible fouling arises from strong adhesion or chemical interactions, such as firmly adsorbed organic compounds, crystallized salts, and fully developed biofilms [75,76]. It should be noted that physical cleaning strategies are ineffective in these cases, and chemical cleaning or even membrane replacement becomes necessary [74,76,77].

Maintaining the separation efficiency of NF membranes requires effective cleaning strategies, which are generally categorized into physical cleaning protocols (e.g., backwashing, static immersion, mechanical scraping, sonication) and chemical cleaning protocols (e.g., acid- and alkali-based cleaning) [74,75,76]. Nevertheless, these cleaning strategies come with considerable limitations since they disrupt the membrane separation process and elevate overall operational costs [75,77]. To overcome these limitations, many studies have focused on engineering and optimizing polymeric NF membranes that exhibit higher flux, tailored morphology, improved hydrophilicity, and enhanced resistance to fouling [78,79]. To date, a variety of advanced modification techniques have been studied, aiming to optimize and enhance the transport, morphological, hydrophilic, and antifouling properties of polymeric NF membranes [79,80]. In general, modification strategies for NF membranes fall into two categories: bulk and surface modification techniques [80,81,82,83]. The main difference between these two methods lies in the type of interactions occurring on the surface or within the membrane bulk [80,81,83].

Up to now, numerous review articles have focused on NF membranes and advances in their development for different applications (Table 2) [25,31,39,84,85,86,87,88,89,90]. In 2023, Maroufi and Hajilary [25] published a detailed review study of various classifications, properties, and applications of NF membranes for water purification process. The review covered the background and the fundamentals of NF membranes, compared their advantages and disadvantages with other membrane processes, and examined various NF membrane materials and the performance of these membranes. Moreover, they emphasized the primary application areas of NF membranes—such as the treatment of surface water, groundwater, and industrial wastewater—while evaluating challenges and future opportunities in advancing the development of more efficient and economical NF membranes. In the same year, Mahmoud and Mostafa [86] published a review study on the application of NF membranes for removing heavy metal ions from contaminated water. They evaluated the influence of nanoparticles and nanofillers on the selectivity, fouling resistance, water flux, and rejection efficiency of these membranes. In addition, the article examined operational parameters, strategies to mitigate fouling, and recent advances in commercially available NF membranes for heavy-metal separation. In 2024, Zheng et al. [39] conducted a review on the crucial factors affecting membrane fouling during water treatment and exploring strategies for predicting the fouling behavior of NF membranes. The article described the mechanisms and progression of membrane fouling, evaluated current progress in machine-learning (ML) methods for fouling prediction, and emphasized the growing role of artificial intelligence (AI) in forecasting future fouling trends. In 2025, Liu et al. [87] conducted a review on the recent advances in positively charged NF membranes, detailing their design, separation mechanisms, and applications in water purification. The study reviewed central application fields, from selective ion separation to wastewater treatment, and discussed performance requirements and challenges. Furthermore, they provided key approaches and future pathways for enhancing membrane efficiency and selectivity. In 2026, Park and Yoon [89] published a review on the utilization of NF membranes for removing per- and poly-fluoroalkyl substances from water and wastewater. The article highlighted the challenges related to poly-fluoroalkyl substances, evaluated the efficiency of NF membranes in eliminating these compounds, and examined the impact of membrane materials and operating conditions on overall separation performance.

**Figure 4:**
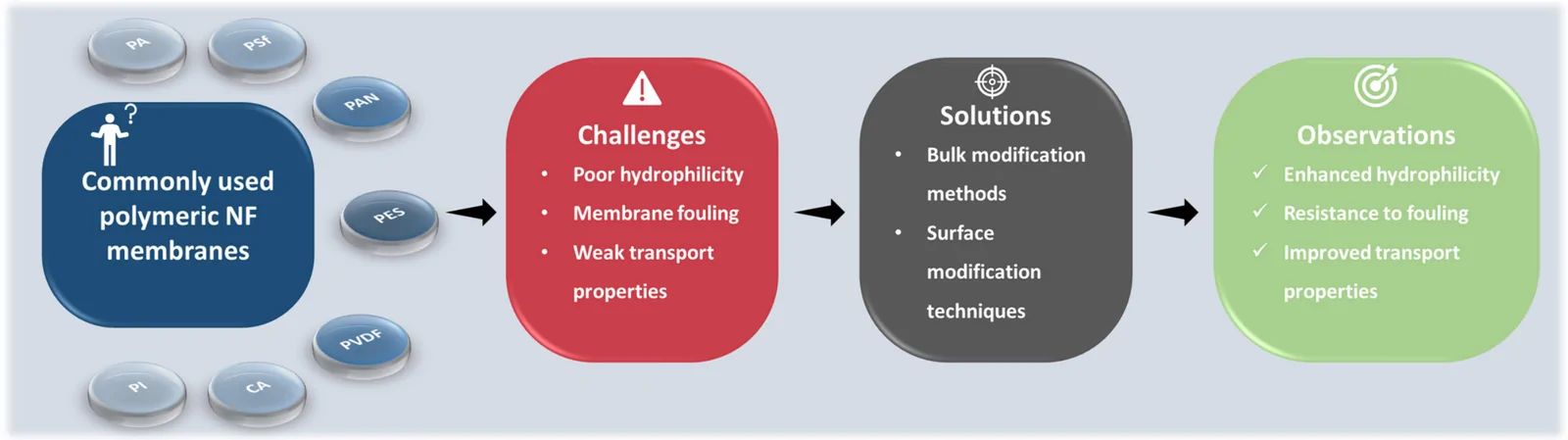
Overview of modification techniques for polymeric NF membranes.

It can be noted that the majority of published review articles have focused on the design, optimization, and enhancement of NF membranes through novel materials and preparation strategies in order to develop these membranes for water and industrial applications [84,85,86,87,88,89,90]. In spite of recent advancements, no review article has systematically covered novel modification methods for improving transport, hydrophilic, and antifouling characteristics of NF membranes (Figure 4). The main aim of this review is to highlight novel bulk modification methods (e.g., polymer blending and nanomaterials incorporation) and surface modification strategies (e.g., physical surface coating, plasma treatment, chemical attachment, layer-by-layer assembly, and interfacial polymerization) for enhancing morphological, hydrophilic, and transport properties of polymeric NF membranes. Moreover, benefits and drawbacks of each method are thoroughly described, accompanied by relevant examples from recent scientific works. Furthermore, the review ultimately provides promising prospects and approaches for designing and tailoring polymeric NF membranes with enhanced durability, transport, and antifouling features. This review follows our earlier publications on MF and UF polymer membranes, which comprehensively addressed recent developments in those fields [21,22]. As the concluding article in this three-part series, the current work concentrates on evaluating the most recent and widely adopted modification strategies that enhance the morphological, hydrophilic, transport, and antifouling properties of NF membranes.

## 2. Methods for Modifying Polymeric NF Membranes

**Figure 5:**
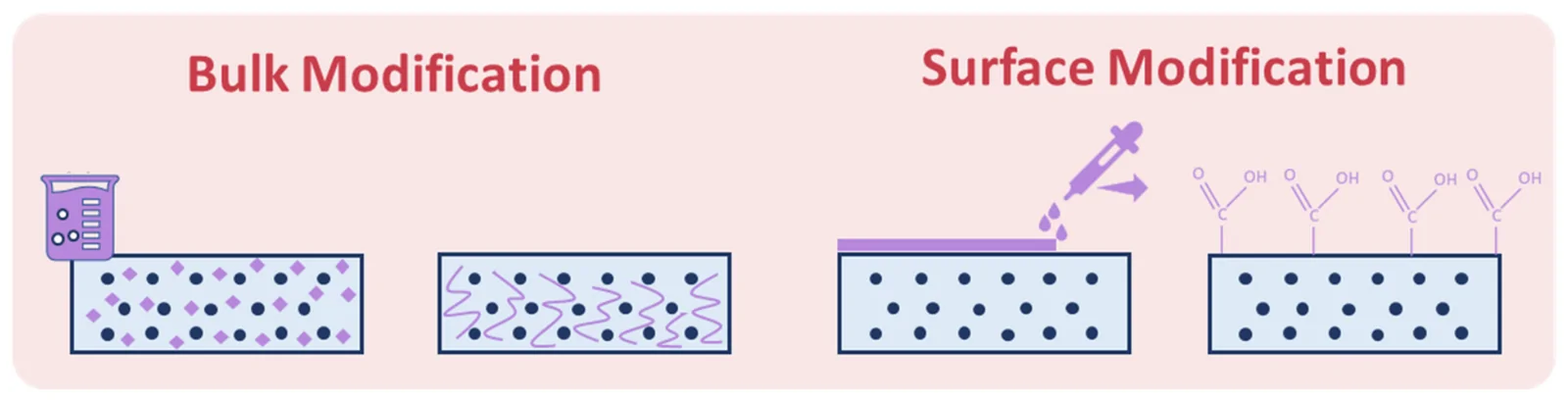
Visual representation of bulk modification and surface modification strategies for polymeric NF membranes.

In general, the modification of polymeric NF membranes can be categorized into two main groups: bulk modification and surface modification methods (Figure 5) [69,70,71,72,73,91,92,93,94,95,96,97,98,99,100,101,102,103,104,105,106,107,108,109,110,111,112,113,114,115,116,117,118]. These two categories are mainly distinguished by the location and extent of the change within the NF membrane [69,70,73,101]. In bulk modification techniques, the entire membrane structure is modified by altering the internal morphology, composition, and transport channels throughout the membrane [92,96,99]. Such changes in membrane properties generally occur during the fabrication stage (e.g., polymer blending and nanomaterial incorporation) [70,73,95,99]. Conversely, surface modification methods mainly focus on tailoring the membrane’s top layer characteristics (e.g., physical surface coating, chemical attachment, layer-by-layer (LbL) assembly, plasma treatment, and interfacial polymerization), while the internal structural properties remain unchanged [100,101,104,108,112]. This review article examines bulk and surface modification strategies, highlighting their benefits and drawbacks along with examples from recently published research articles.

### 2.1. Bulk Modification Methods

In this type of modification, the membrane’s internal structure and composition (especially the support layer or the polymer matrix) are altered rather than only its surface [73,93]. Therefore, bulk modification methods are also known as internal or matrix modification techniques [91,92,98]. These methods—such as polymer blending and nanoparticle incorporation—can significantly influence the morphology (i.e., porosity and pore size distribution), hydrophilicity, mechanical stability, transport, and antifouling properties of NF membranes [93,98,99]. Such modifications are commonly used owing to their benefits, including operational simplicity, affordability, scalability, reversibility, and low environmental impact [70,73,98]. However, such modified NF membranes suffer from a lack of stable hydrophilicity, transport performance, and antifouling properties as the interactions in the bulk are physical, causing leaching of the additive from the membrane matrix [69,91,92,99].

#### 2.1.1. Polymer Blending

Among physical modification strategies for polymeric NF membranes, polymer blending is one of the most commonly applied techniques [69,93]. The general protocol for membrane modification in this approach involves dissolving and mixing multiple polymers in the same solvent [69,70,91,92]. The non-solvent induced phase separation (NIPS) technique is usually used for the preparation of polymeric NF membranes in this method. The main objective of this method is to improve the hydrophilicity and morphology of well-known polymeric NF membranes, such as polyvinyl chloride (PVC), PVDF, PES, and PSf, by adding hydrophilic polymers, including polyvinyl alcohol (PVA), chitosan (CS), polyethylene glycol (PEG), and polyvinylpyrrolidone (PVP) [69,70,73,91,92,93]. It should be highlighted that some polymers, such as PEG and PVP, can contribute to enhanced membrane hydrophilicity, which is attributed to hydrophilic groups in their structure, while simultaneously improving membrane morphology (i.e., both surface and structural porosity) by functioning as pore formers during the membrane formation step [70,91,92]. Despite the advantages of the polymer blending method, namely scalability, operational simplicity, cost-effectiveness, and adaptability, it is hindered by a few limitations, such as insufficient polymer dispersion and compatibility [73,93].

Hardian et al. [73] developed cellulose/chitosan (CS) NF membranes containing various concentrations of CS (i.e., 0 (M_0_), 10 (M_1_), 18 (M_2_), and 25 (M_3_) wt.%) by using a polymer blending technique. The main objective of that research was to introduce new type of NF membranes containing excellent transport properties derived from bio-based materials which have potential for recycling. It was found that M_0_ membrane showed higher water permeance (95.3 L·m^−2^·h^−1^·bar^−1^) than blend membranes (38.1 to 52.1 L·m^−2^·h^−1^·bar^−1^), while blend membranes exhibited higher oil removal efficiency (73.8 to 98.6%) than pure cellulose membrane (44.0%). However, it was found that the oil–water permeance of M_3_ membrane significantly reduced from 48.6 to 31.6 L·m^−2^·h^−1^·bar^−1^ over time, owing to the membrane fouling and pore blockage, while the oil removal efficiency remained stable over time (97%). Such observations confirm that by blending cellulose with chitosan, NF membranes possessing excellent permeance, rejection performance, and mechanical stability were obtained.

Cheng et al. [69] prepared NF membranes by blending PES with sulfonated PSf. Four PES/sPSf membranes with different blend ratios (PES:sPSf = 84:16 (M_0_), 72:24 (M_1_), 68:32 (M_2_), and 60:40 (M_3_)) were fabricated via the NIPS method. The scanning electron microscopy (SEM) images showed that increasing the concentration of sPSf transformed the membrane morphology, changing from a finger-like structure to a sponge-like one, due to the increased viscosity of the polymer solution (from 5060 to 6120 mPa·s). Moreover, the PEG rejection increased when raising the content of sPSf (M_0_ → M_3_) from 58 to 98%, while an opposite trend was seen for water permeance, with the M_0_ membrane demonstrating the highest value (35.5 L·m^−2^·h^−1^·bar^−1^). The blend PES/sPSf membranes exhibited excellent levofloxacin (LVFX) rejection (between 96 and 99%), while showing poor rejection of magnesium chloride (MgCl_2_) (less than 5%). These observations indicate that applying a polymer blending strategy can noticeably enhance the morphology, rejection performance, and transport properties of NF membranes.

**Figure 6:**
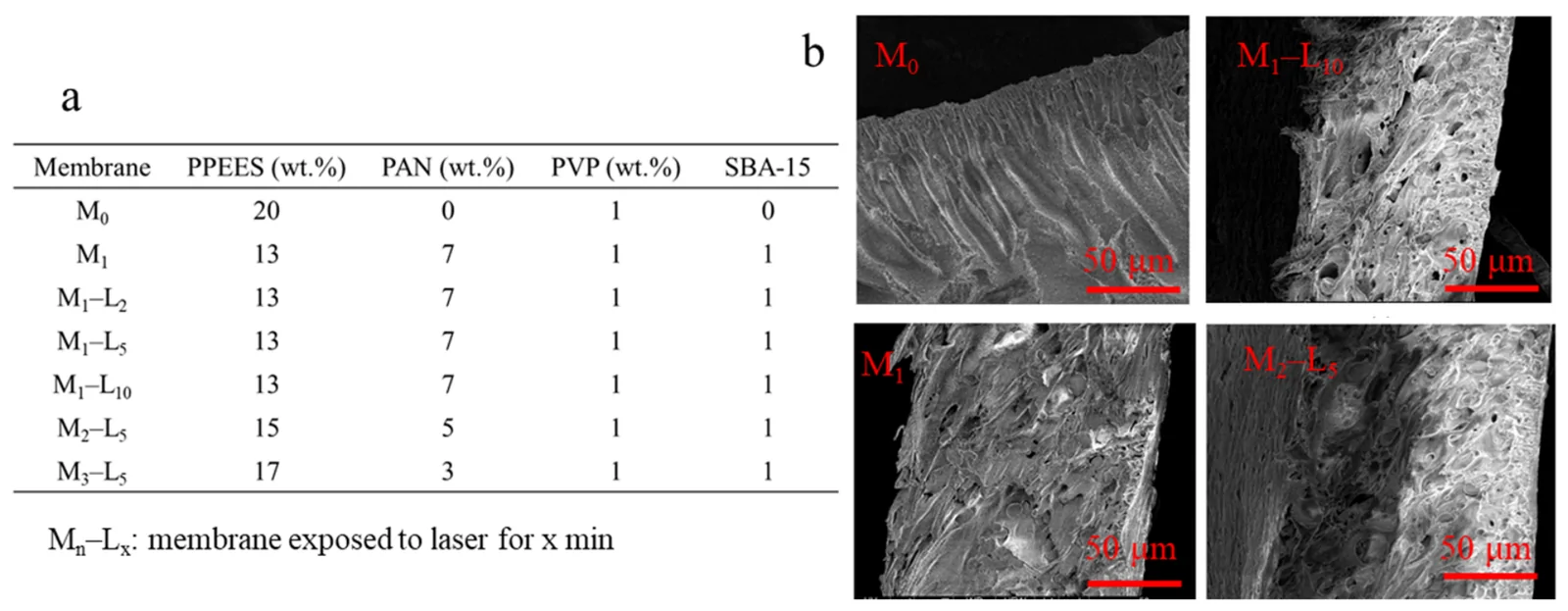
(**a**) Membrane compositions and (**b**) SEM micrographs of PPEES-based NF membranes (adapted from Paun et al., 2022) [70].

In another study, Paun et al. [70] prepared hybrid NF membranes by blending poly(1,4-phenylene ether ether sulfone) (PPEES), PAN, PVP, and mesoporous silica (SBA-15) using a phase inversion technique. Several membranes containing different concentrations of PAN were prepared, and some of the samples were subjected to laser treatment (Figure 6a). The cross-sectional SEM images showed that the structural morphology of the pure PPEES membrane shifted from a finger-like morphology to a sponge-like one owing to the addition of PAN (Figure 6b). Moreover, the hybrid membranes demonstrated lower WCA values (between 40 and 52°) than the pure PPEES membrane (~68°), confirming the enhanced hydrophilicity of the hybrid membranes due to the hydrophilic properties of PAN. The hybrid membranes exhibited higher water permeance (from 1.5 to 13.3 L·m^−2^·h^−1^·bar^−1^) and polyphenol rejection values (up to 97.8%) than the M_0_ membrane (1.4 L·m^−2^·h^−1^·bar^−1^ and 68.8%, respectively). The observation reveals that the morphological, hydrophilic, and transport properties of the hybrid membranes are improved by adding PAN, owing to the presence of polar nitrile groups in the structure of PAN.

Dehban et al. [71] developed a new type of NF membrane by blending poly(phenyl sulfone) (PPSU) with PES. Several PPSU/PES membranes containing different blend ratios (PPSU:PES = 0:100 (M_0_), 25:75 (M_1_), 50:50 (M_2_), 75:25 (M_3_), and 100:0 (M_4_)) were fabricated using a combined vapor-induced phase separation (VIPS)–NIPS technique. It was observed that the WCA values slightly increased with rising PPSU content, from ~73° to ~75°, indicating that PES is slightly more hydrophilic than PPSU because of the presence of ether groups in the PES structure. It was found that the pure water permeance of the blend membranes was higher than that of pure PES and PPSU membranes and the highest value was reported for the M_2_ membrane (~37.8 L·m^−2^·h^−1^·bar^−1^), while the pure membranes (M_0_ and M_4_) showed higher Rose Bengal (RB) rejection (~91%) compared to the M_2_ membrane (~62%). Such research illustrates that, by blending two different polymers, membranes containing different hydrophilicity, morphology, rejection performance, and transport properties can be obtained.

Bagheripour et al. [72] fabricated NF membranes by blending PES and sulfonated PVC. Moreover, PVP and dimethylacetamide (DMAc) were used as the pore former and solvent, respectively. Five PES/sPVC membranes possessing different blend ratios (PES:sPVC = 100:0 (M_0_), 95:5 (M_1_), 90:10 (M_2_), 85:15 (M_3_), and 80:20 (M_4_)) were prepared via the NIPS method. It was found that water content was enhanced by increasing the amount of sPVC, indicating an improvement in the membrane hydrophilicity owing to the hydrophilic nature of sPVC. Additionally, the membrane porosity of PES/sPVC membranes increased (between ~82–87%) compared to the pristine PES membrane (~72%) with the addition of sPVC, and the highest value was reported for the M_1_ membrane. It was observed that the blend membranes showed higher pure water permeance and sodium sulfate (Na_2_SO_4_) rejection values (up to 34.1 L·m^−2^·h^−1^·bar^−1^ and 67%, respectively) than the pristine membrane (0.5 L·m^−2^·h^−1^·bar^−1^ and 53%, respectively), owing to the improved membrane hydrophilicity and morphology. That study reveals better rejection performance and transport properties for the blend PES/sPVC membranes compared with the pristine PES membrane owing to improved membrane hydrophilicity and morphology resulting from sPVC addition.

In another study, Yahya et al. [91] developed polymeric NF membranes from PPSU and PES in order to eliminate 4-nitrophenol (4-NP) from water. Five polymeric blend membranes with different concentrations of PES (0, 6, 7, 8, and 9 wt.%) but the same concentration of PPSU (20 wt.%) were prepared using the NIPS method. It was found that the blend membranes demonstrated lower WCA values (70–75°) than the pure PPSU membrane (77°), confirming improved membrane hydrophilicity. In addition, the blend membranes showed higher 4-NP permeance (up to ~3.6 L·m^−2^·h^−1^·bar^−1^) and rejection (up to 98%) in comparison with the pure membrane (~2.5 L·m^−2^·h^−1^·bar^−1^ and 81%, respectively). These observations show that by using a polymer blending technique, hydrophilicity, rejection performance, and transport properties of polymeric NF membranes can be improved considerably.

Liang et al. [92] prepared a new type of loose NF membranes by blending PES and hydroxylated PES using the NIPS method. In that study, butyl decarbonate (BDC) was used as a reactive porogen, which decomposed to form CO_2_ nanobubbles during membrane preparation at different temperatures (20–70 °C), leading to control of the membrane morphology. The blend PES/PES–OH membranes showed high porosity (between 51.7 and 84.5%), and increasing the heat-treatment temperature led to enhanced porosity. Moreover, the blend membranes exhibited excellent pure water permeance (~200 L·m^−2^·h^−1^·bar^−1^), dye solution permeance (~175 L·m^−2^·h^−1^·bar^−1^) and direct black (DB38) rejection (~99%), while showing poor NaCl rejection (<5%). That research illustrates that the transport properties and the removal rate of PES/PES–OH membranes were enhanced owing to improved hydrophilicity (the presence of PES–OH) and morphology (the addition of BDC and heat-treatment stage) of membranes.

In another study, Yao et al. [93] developed new hollow fiber NF membranes by blending carboxylated polyethersulfone (PES-COOH) with PSf using a NIPS technique. Five PSf/PES–COOH membranes with various amounts of PES–COOH (0.0 (M_0_), 0.8 (M_1_), 1.6 (M_2_), 4.8 (M_3_), and 8.0 (M_4_) wt.%) and the same concentration of PEG (3.0 wt.%, pore-forming agent) were prepared. The cross-sectional images illustrated two structural morphologies of the prepared membranes: a finger-like structure in the top and bottom layers and a sponge-like structure in the center of membranes. It was found that increasing the amount of PES–COOH led to an increase in the size of finger-like pores. Moreover, blend membranes possessing a small amount of PES–COOH exhibited higher water permeance (up to 230 L·m^−2^·h^−1^·bar^−1^) and Congo red (CR) rejection (>97%) values than pure PSf membrane (~135 L·m^−2^·h^−1^·bar^−1^ and ~93%, respectively). The antifouling test showed that the CR/NaCl solution permeance decreased by around 81% (after 2 h), while the permeance was restored to 91% of its initial value after the cleaning process, indicating the promising antifouling properties of PSf/PES–COOH membranes. Such observations confirm that the polymer blending technique can significantly enhance the morphology, transport, and antifouling properties of polymeric NF membranes.

#### 2.1.2. Incorporation of Nanoparticles

The incorporation of nanoparticles (NPs) is considered a promising approach to enhance hydrophilicity, morphology, transport, antifouling, and antibacterial properties of polymeric NF membranes [94,97,99]. By enhancing the hydrophilicity of NF membranes, these additives contribute to a considerable reduction in fouling tendencies [96,98]. Moreover, introducing NPs may improve mechanical and thermal stability of NF membranes [95,97]. These membranes are also known as mixed matrix membranes (MMMs), which can significantly overcome the well-known trade-off in NF polymeric membranes, where rising permeate flux usually decreases selectivity [98,99]. In MMMs, these two properties can be improved simultaneously [94,96,99]. However, such modified composite membranes mainly suffer from the leaching of nanomaterials over time, causing instability of hydrophilic, transport, and antifouling properties of these membranes [95,96]. To overcome this disadvantage, such additives should be grafted onto the polymer chains or through membrane crosslinking. To date, a broad spectrum of NPs—such as titanium dioxide (TiO_2_), zinc oxide (ZnO), silicon dioxide (SiO_2_), graphene oxide (GO), silver (Ag), iron (III) oxide (Fe_2_O_3_), cerium (IV) oxide (CeO_2_), chitosan (CS), and zeolite NPs—have been employed to improve the rejection efficiency and transport characteristics of polymeric NF membranes [94,95,97,98,99].

**Figure 7:**
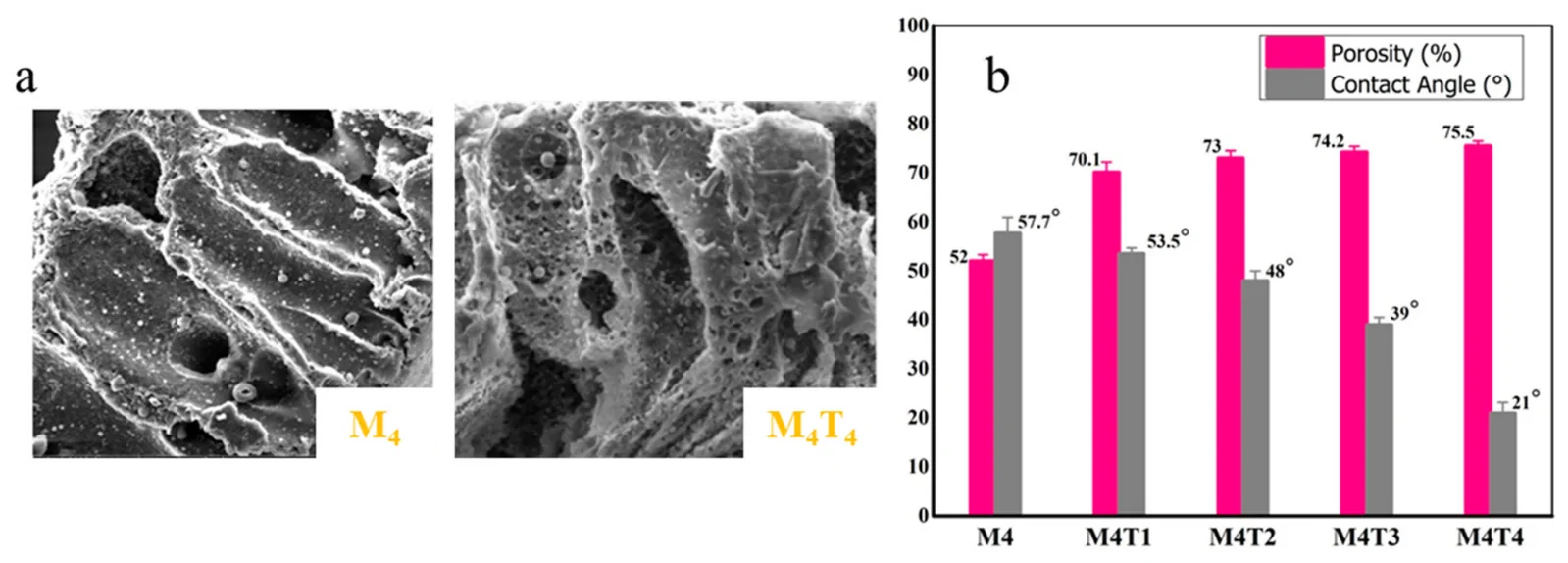
(**a**) SEM micrographs and (**b**) porosity and WCA of CA/PES/TiO_2_ membranes (adapted from Batool et al., 2021) [94].

Batool et al. [94] developed MMMs by incorporating TiO_2_ NPs into a polymer blend containing CA and PES for water desalination. Five MMMs containing different concentrations of TiO_2_ NPs (0.0 (M_4_), 0.5 (M_4_T_1_), 1.0 (M_4_T_2_), 1.5 (M_4_T_3_), and 2.0 (M_4_T_4_) wt.%), along with the same amounts of CA and PES (74 and 18.5 wt.%, respectively), were fabricated using a NIPS technique. It was found that increasing the concentration of TiO_2_ in the polymer blend led to the formation of macrovoids and an increased membrane porosity (from 52.0 to 75.5%) (Figure 7a,b). Moreover, MMMs showed lower WCA values (between 21 and 53.5°) than the blend CA/PES membrane (57.7°), indicating enhanced hydrophilicity (Figure 7b). Furthermore, modified CA/PES/TiO_2_ membranes exhibited higher water permeance (between 12.6 and 14.9 L·m^−2^·h^−1^·bar^−1^) and NaCl rejection (between 68.0 and 76.8%) compared to the pristine CA/PES membrane (10.8 L·m^−2^·h^−1^·bar^−1^ and ~64.0%, respectively), due to improved hydrophilicity resulting from the incorporation of TiO_2_ NPs. These results indicate that the incorporation of TiO_2_ NPs into the blend polymer contributed to enhanced hydrophilicity, morphology, and transport properties of the NF membranes.

In another study, Purushothaman et al. [95] fabricated composite membranes possessing poly(ether ether sulfone) (PEES) and ZnO NPs. Six MMMs with different amounts of ZnO NPs (0.0, 0.2, 0.4, 0.6, 0.8, and 1.0 wt.%) were prepared via the NIPS technique. Morphological studies showed higher mean pore size (4.7–6.4 nm) and porosity (18.2–28.7%) values for PEES/ZnO membranes compared with the pure PEES membrane (3.2 nm and 14.1%, respectively). In addition, PEES/ZnO membranes demonstrated higher water permeance (5.0–7.7 L·m^−2^·h^−1^·bar^−1^) and HA rejection (94.4–98.0%) than the pure PEES membrane (4.0 L·m^−2^·h^−1^·bar^−1^ and 97.2%, respectively), owing to enhanced membrane morphology and hydrophilicity resulting from ZnO addition. The antifouling test showed a higher FRR for modified membranes (between 80.6 and 92.4%) compared to the unmodified membrane (44.6%), confirming improved antifouling properties of MMMs. Such observations indicate that the incorporation of ZnO NPs can significantly improve the morphology, hydrophilicity, rejection performance, transport, and antifouling properties of PEES membrane.

**Figure 8:**
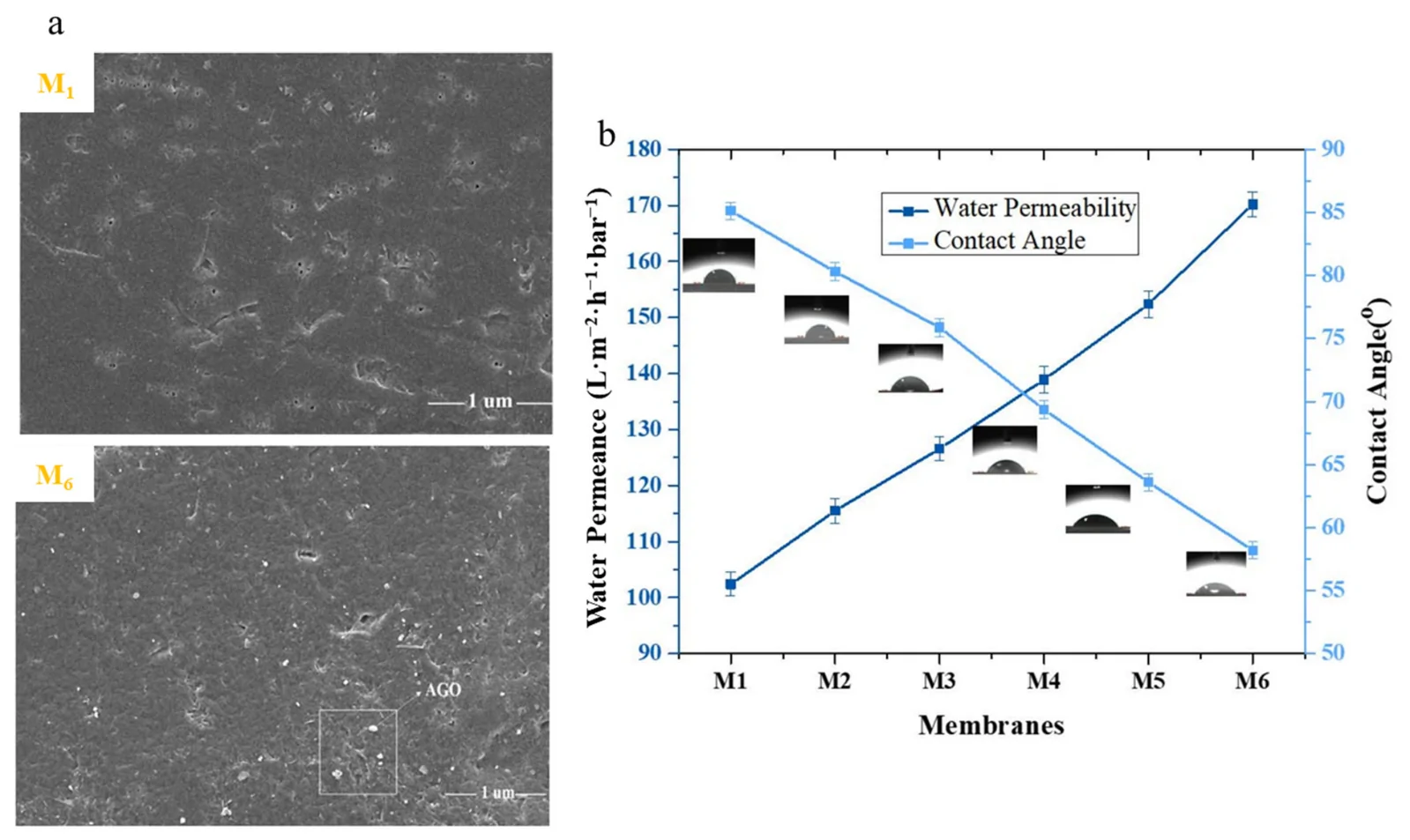
(**a**) SEM micrographs and (**b**) water permeance and WCA of PVDF/AGO membranes (adapted from Ahmad et al., 2022) [96].

Ahmad et al. [96] developed a new set of MMMs comprising PVDF and aminated graphene oxide (AGO) using the NIPS technique. In that study, several membranes were prepared with various amounts of AGO (0.0 (M_1_), 0.2 (M_2_), 0.4 (M_3_), 0.6 (M_4_), 0.8 (M_5_), and 1.0 (M_6_) wt.%) to evaluate the influence of AGO concentration on the PVDF membrane properties. SEM images showed a uniform distribution of AGO NPs in the membrane structure (Figure 8a). Moreover, MMMs demonstrated enhanced porosity (88.5–90.7%) in comparison with the pure PVDF membrane (87.7%), confirming improved membrane morphology owing to AGO incorporation. It was found that the WCA of the pure PVDF membrane decreased after NP incorporation (from 85° to 58°), indicating enhanced membrane hydrophilicity (Figure 8b). Additionally, PVDF/AGO membranes exhibited higher water permeance (115.5–170.2 L·m^−2^·h^−1^·bar^−1^) compared to the pure PVDF membrane (102.4 L·m^−2^·h^−1^·bar^−1^), due to the improvement in membrane morphology and hydrophilicity resulting from AGO incorporation (Figure 8b). All observations indicate that AGO incorporation led to enhanced hydrophilic, morphological, and transport features of the PVDF membrane.

In another research work, Bashir et al. [97] prepared composite membranes containing PES and Ag NPs. Five membranes composed of different contents of Ag NPs (0.00, 0.25, 0.50, 0.75, and 1.00 wt.%) were fabricated via the NIPS technique. SEM images demonstrated that Ag NPs acted as a pore-forming agent, such that more porous membranes were obtained with increasing Ag NPs concentration. It was seen that the addition of Ag NPs led to improving membrane hydrophilicity. Moreover, the composite membranes exhibited higher pure water permeance (up to 36 L·m^−2^·h^−1^·bar^−1^) than the unmodified PES membrane (11 L·m^−2^·h^−1^·bar^−1^), because of the improved membrane morphology and hydrophilicity resulting from Ag NPs addition. The composite PES/Ag membranes also showed higher MB (90–98%), RB (53–85%), and CR (89–99%) rejection values compared to the pure PES membrane (41%, 42%, and 65%, respectively). These findings indicate that the incorporation of Ag NPs resulted in NF membranes possessing enhanced morphology, hydrophilicity, rejection efficiency, and transport properties.

**Figure 9:**
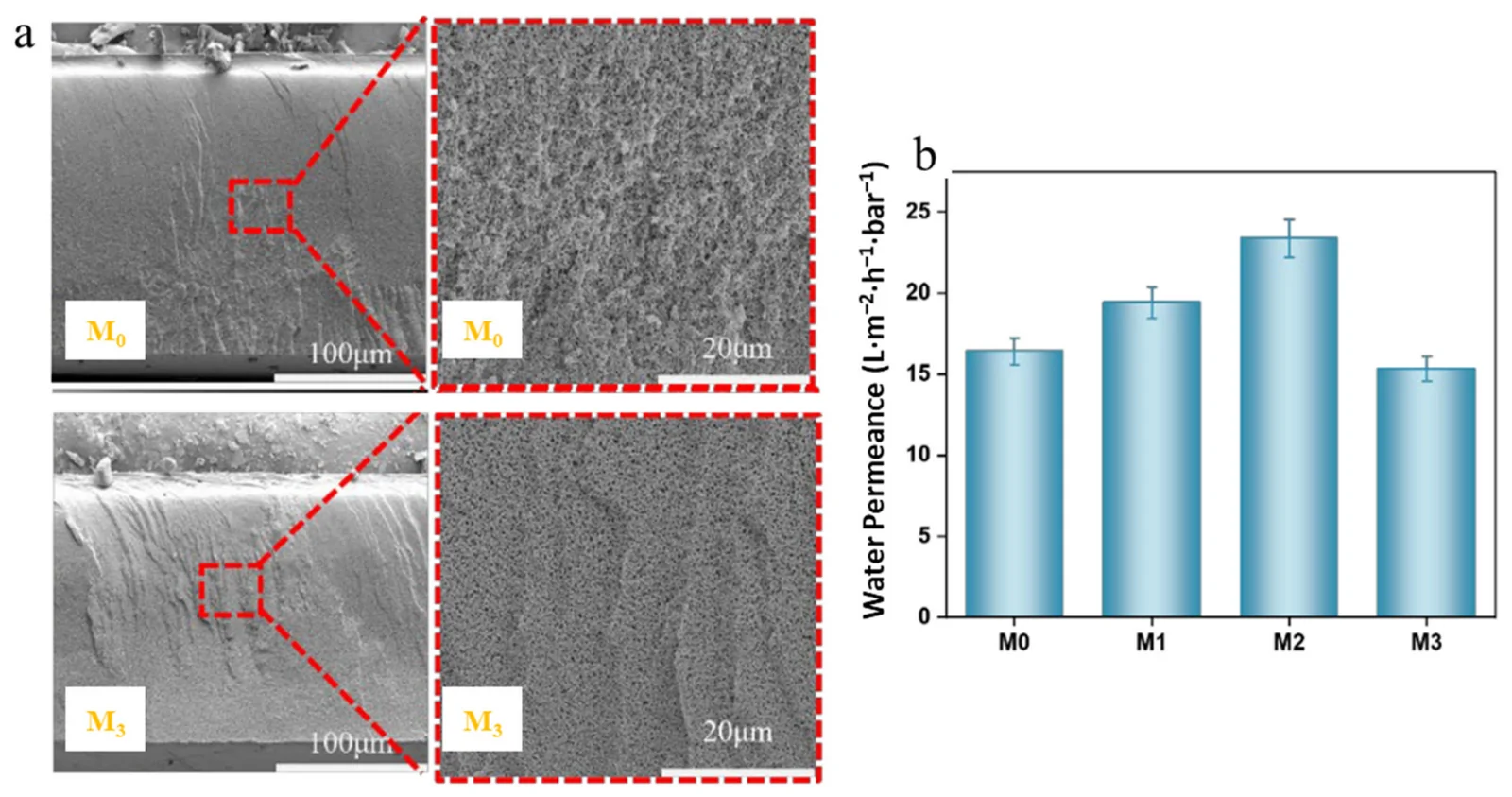
(**a**) SEM micrographs and (**b**) pure water permeance of PES/sPES/Ti_3_C_2_T_X_ membranes (adapted from Meng et al., 2025) [98].

Meng et al. [98] developed polymeric NF membranes from PES and sulfonated PES, and to enhance the characteristics of membrane, monolayer titanium carbide nanosheets (Ti_3_C_2_T_X_) were incorporated into the polymer blend. Four PES/sPES/Ti_3_C_2_T_X_ membranes with different mass ratio of Ti_3_C_2_T_X_ (0, 0.1, 0.3, and 0.5%) but the same concentrations of PES and sPES were prepared using the NIPS method. The morphological studies revealed that by increasing the Ti_3_C_2_T_X_ amount, the apparent pore size of the membranes initially reduced (from M_0_ to M_2_) and then increased (M_3_) (Figure 9a). Such observation can be associated with the dual regulatory impacts of the nanosheets on the phase inversion process. The M_2_ membrane exhibited the highest pure water permeance of ~24 L·m^−2^·h^−1^·bar^−1^ owing to its improved hydrophilicity and morphology (i.e., higher porosity) (Figure 9b). In addition, PES/sPES/Ti_3_C_2_T_X_ membranes (in particular, M_1_ and M_2_) showed enhanced salt and dye rejection performance compared with the PES/sPES membrane. M_1_ and M_2_ membranes also showed higher FRR values (53.1–54.8%) compared with M_0_ (49.5%), confirming their better antifouling properties. Such observations indicate that the addition of nanomaterials at an optimum concentration can considerably influence the morphology, transport, and antifouling properties of polymeric NF membranes.

In another study, Hosseini et al. [99] developed MMMs containing PES and Fe_3_O_4_-PVP composite NPs. Six NF membranes composed of varying amounts of Fe_3_O_4_-PVP NPs (0.00 (M_0_), 0.05 (M_1_), 0.10 (M_2_), 0.50 (M_3_), 1.00 (M_4_), and 2.00 (M_5_) wt.%) were fabricated via the NIPS technique. The cross-sectional SEM images showed that the addition of composite NPs led to the formation of larger finger-like channels. Furthermore, MMMs showed enhanced hydrophilicity, as they exhibited lower WCA values (61.8–50.5°) in comparison with M_0_ (65.2°) due to the excellent hydrophilic properties of Fe_3_O_4_-PVP NPs. Additionally, the composite membranes exhibited higher water permeance (up to 2.0 L·m^−2^·h^−1^·bar^−1^) and salt rejection values (up to 90%) than the M_0_ (0.6 L·m^−2^·h^−1^·bar^−1^ and 82%, respectively) because of their improved morphology and hydrophilicity resulting from the incorporation of Fe_3_O_4_-PVP NPs. MMMs also showed higher FRR ratios (between 70.1% and 89.5%) than the unmodified PES membrane (46.2%), confirming improved antifouling properties. These findings indicate that the addition of NPs―even at low concentrations―can significantly improve the hydrophilicity, morphology, rejection performance, transport, and antifouling of polymeric NF membranes. Table 3 provides a summary regarding the bulk modification methods (i.e., polymer blending and NP incorporation) for polymeric NF membranes.

### 2.2. Surface Modification Methods

In surface modification techniques, only the properties of the outer layers of NF membranes are altered for enhancing the interactions with the feed stream without significantly affecting the internal membrane structure [100,101,113]. In general, the main objective of such modifications is to improve key features, including hydrophilicity, surface morphology, surface charge, rejection efficiency, permeance, and antifouling properties [101,107,112]. The most commonly used approaches include physical surface coating, chemical attachment, layer-by-layer (LbL) assembly, plasma treatment, and interfacial polymerization (IP) [100,107,108]. Even though these modifications can considerably enhance the NF membrane separation performance, their main disadvantage is the gradient of properties, referring to distinct properties in different regions of the membrane [101,108]. However, these methods offer the possibility for precise surface engineering and localization of desirable properties [109,110,111,112,113,115,116].

#### 2.2.1. Physical Surface Coating

Physical surface coating is one of the most commonly used surface modification methods, mainly thanks to its process simplicity [101,103]. In this strategy, a thin active layer of material (e.g., a hydrophilic polymer or nanomaterial) is coated onto the membrane surface through weak physical interactions (e.g., electrostatic interactions and van der Waals forces) [100]. Such surface-modified membranes are usually prepared via dip coating, spray coating, or spin coating techniques [101,102]. This approach can significantly improve membrane hydrophilicity and selectivity without changing the internal properties of the polymeric NF membrane [103]. Furthermore, these membranes generally show enhanced antifouling properties, which leads to reduced cleaning costs. Simplicity, cost-effectiveness, and versatility in material selection are the most important benefits of this method [100,102]. However, due to the lack of strong chemical bonds, the coated layer can be leached out over time, causing instability in hydrophilic, transport, and antifouling properties, which the severity of this issue can vary depending on the operating conditions, including pressure, temperature, and filtration type [100,101,102,103].

Huang et al. [100] prepared the surface-modified NF membranes by coating polydopamine (PDA) on the surface of NF90 commercial membranes. The surface modification process was carried out during different coating periods: 0.0 h (M_0_), 0.5 h (M_1_), 1.0 h (M_2_), 2.0 h (M_3_), and 4.0 h (M_4_). The WCA measurements showed that the introduction of the PDA layer on the NF90 membrane resulted in decreasing WCA values. By increasing the PDA deposition time from 0 h to 4 h, the WCA decreased significantly (from 68.4 to 47.3°). Such an observation confirms the membrane hydrophilicity improvement owing to the excellent hydrophilic properties of PDA (mainly due to the presence of –OH and –NH_2_). NF90/PDA membranes showed higher NaCl rejections (up to 93.2%) than pure NF90 membrane, whereas they exhibited lower pure water permeance compared to the unmodified membrane (9.84 L·m^−2^·h^−1^·bar^−1^). These findings reveal that there is a trade-off between deposition time and membrane properties, where excessive PDA deposition led to reduced water permeance. Therefore, optimizing the modification process is necessary to obtain polymeric NF membranes with ideal hydrophilicity and transport properties.

**Figure 10:**
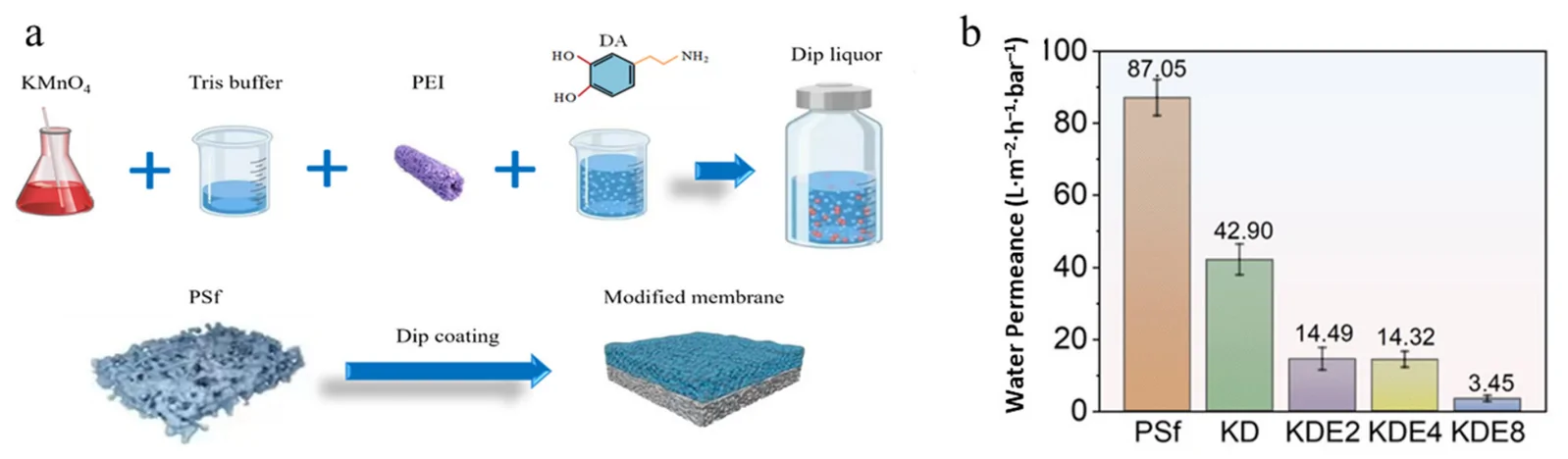
(**a**) Coating schematic diagram and (**b**) water permeance of PSf-based NF membranes (adapted from Li et al., 2025) [101].

Li et al. [101] developed a new polymeric NF membrane by coating a layer of activated polydopamine (A-PDA), oxidized with potassium permanganate (KMnO_4_), together with polyethyleneimine (PEI) onto a PSf porous support (Figure 10a). Several surface-modified membranes were prepared by dipping them into various solutions containing different concentrations of PEI (0 (KD), 2 (KDE2), 4 (KDE4), 8 (KDE8), and 12 (KDE12) g·L^−1^) along with the same concentrations of KMnO_4_ (0.666 g·L^−1^) and PDA (2 g·L^−1^). It was found that the pristine PSf membrane showed higher water permeance (~87.1 L·m^−2^·h^−1^·bar^−1^) than surface-modified membranes (3.5–42.9 L·m^−2^·h^−1^·bar^−1^), due to the aggregate pore resistance formation on the surface of the modified membranes (Figure 10b). However, the surface-modified membranes exhibited higher NaCl (up to 47.0%), MgCl_2_ (up to 57.2%), Na_2_SO_4_ (up to 55.8%), MgSO_4_ (68.2%), CR (up to 99.7%), and MB (up to 88.5%) rejection performance than pristine PSf membrane. These findings reveal that, although the surface-modified membranes showed enhanced rejection performance, they demonstrated lower water permeance values than the pristine PSf membrane. Therefore, a balance should be maintained in this modification method to obtain a modified membrane with optimal properties.

Babu et al. [102] prepared thin film composite (TFC) membranes with a physical coating of PVA on the PES support and evaluated their potential for dye and salt removal from textile wastewater. For this, the porous PES membrane was initially immersed into a water/PVA solution (PVA concentration: 0.1, 0.5, 1.0, and 1.5%). Subsequently, the coated membrane was immersed into the crosslinking solution—containing maleic acid (crosslinker), water, and H_2_SO_4_—to create stable PVA–PVA networks. The surface-modified membranes showed excellent hydrophilicity (WCA: 29.5–54.9°); however, increasing the PVA concentration brought about a higher WCA. This phenomenon is attributed to the formation of a denser and more highly crosslinked layer, leading to a decrease in the availability of hydroxyl groups at the surface of PES/PVA membranes. It was found that increasing the concentration of PVA (from 0.1 to 1.5%) led to a water permeance decline (from 8.0 to 2.5 L·m^−2^·h^−1^·bar^−1^) owing to two reasons: 1. an increase in active layer thickness, and 2. a decrease in hydrophilicity. In contrast, surface-modified membranes containing higher PVA concentrations showed higher rhodamine rejection, where the maximum value was observed for PES/PVA_1.5%_ (~93%). Therefore, PES/PVA_1.0%_ membrane was selected as the optimal membrane, demonstrating desirable water permeance and dye rejection (3.0 L·m^−2^·h^−1^·bar^−1^ and 89%, respectively). Such observations indicate that by depositing the optimum thickness of PVA layer, membranes with desirable hydrophilicity, water permeance, and rejection can be achieved.

In another study, Mahalingam et al. [103] developed polyetherimide (PEIm) hollow fiber membrane via a spinning process and in order to enhance the chemical stability, crosslinking process was performed using hexamethylene diamine (HMD) as a crosslinking agent. Subsequently, different volumes of GO (2, 4, and 6 mL) were deposited on the surface of the crosslinked PEIm membranes by a spray coating technique to evaluate the influence of GO layer thickness on the membrane characteristics. The pure PEIm membrane showed a water permeance of 179 L·m^−2^·h^−1^·bar^−1^ and this value reduced (135 L·m^−2^·h^−1^·bar^−1^) after crosslinking reaction due to changes in pore structure. Additionally, the surface-modified PEIm/GO membranes showed lower water permeance than the pure membrane, and water permeance further decreased from 40 to 12 L·m^−2^·h^−1^·bar^−1^ with increasing GO layer thickness. A similar trend was observed for acetone permeation behavior. Furthermore, surface-modified membranes demonstrated promising RB rejection performance (between 80 and 91%) as increasing GO volume resulted in enhanced RB rejection. These observations confirm that the rejection performance of polymeric NF membranes can be enhanced by the surface coating method. However, excessive deposition can considerably reduce permeance. As a result, synergy between the thickness of coating layer and membrane transport properties should be considered.

#### 2.2.2. Chemical Attachment

Unlike physical surface coating technique, in this method, the modified layer is strongly linked to the membrane support through covalent bonds [104,105]. Therefore, such modified membranes can exhibit stable and efficient selectivity and antifouling properties [106,107]. This modification is usually performed through crosslinking or grafting processes, where the desirable material (i.e., polymer chain or functional group) is chemically linked to the membrane surface [104,106]. However, there are some disadvantages to this method, such as reduced permeance and high cost [105,107]. Moreover, this modification method is a rather complex and tricky process in a way that excessive grafting and crosslinking can result in pore blockage and subsequent problems, such as severely reduced permeance and rapid membrane fouling [105]. As a result, process optimization is necessary to strike a balance among transport properties (i.e., permeance and selectivity) [104,106].

**Figure 11:**
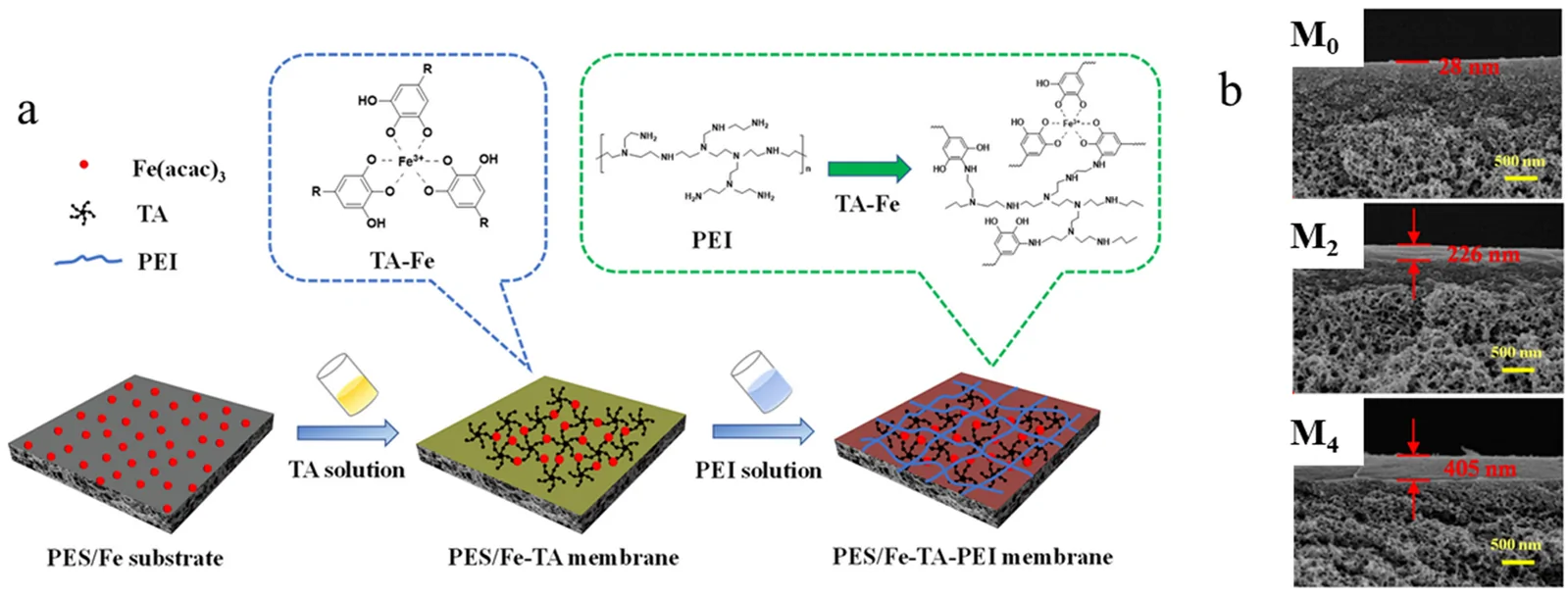
(**a**) Schematic diagram of membrane preparation and (**b**) SEM micrographs of PES/Fe-TA-PEI membranes (adapted from Liu et al., 2021) [104].

Liu et al. [104] developed a loose PES/Fe-based NF membrane by using a grafting method. The PES/Fe membrane was initially fabricated using the NIPS technique. In the next step, the PES/Fe support was immersed in tannic acid (TA) solution to allow TA to bind to Fe metal–polyphenol coordination complexes. Subsequently, the PES/Fe-TA membrane was immersed in solutions with different concentrations of PEI (0.0 (M_0_), 0.5 (M_1_), 1.0 (M_2_), 1.5 (M_3_), and 2.0 (M_4_) g·L^−1^) to chemically link PEI to TA via a Michael addition strategy (Figure 11a). The cross-sectional SEM images showed that increasing the PEI concentration led to an increase in the thickness of the TA-PEI layer from 28 to 405 nm (Figure 11b). It was found that increasing the PEI concentration led to a decline in water permeance from 104.4 to 15.6 L·m^−2^·h^−1^·bar^−1^, while the Alcian Blue 8GX (AB 8GX) rejection enhanced from 74.6 to 98.7%. Therefore, M_2_ was selected as the ideal membrane containing optimal pure water permeance and rejection performance. Furthermore, the M_1_ membrane showed promising dye/salt permeance and dye rejection (29.8–32.3 L·m^−2^·h^−1^·bar^−1^ and 83.5–99.3%, respectively), whereas it exhibited poor NaCl rejection (2.9–4.2%). These observations confirm that the rejection efficiency of surface-modified membranes can be improved. However, overloading can dramatically decrease permeance. Hence, an optimal membrane with desirable transport and rejection performance should be designed.

Hu et al. [105] developed a new polymeric crosslinked NF membrane using a chemical surface modification method. In that study, a porous support was prepared by blending poly(styrene-maleic anhydride) (SMA) with PES. Subsequently, a thin layer of PVA was crosslinked (through esterification) onto the PES/SMA support. The pure PES membrane showed a WCA of 86°, while this value was slightly reduced (78°) for PES/SMA membrane because of the presence of anhydride groups. However, after surface modification, the WCA was significantly reduced (54°) owing to the excellent hydrophilic properties of PVA (resulting from –COOH and –OH polar groups). The surface-modified membrane showed stable permeance (~11 L·m^−2^·h^−1^·bar^−1^, for 100 ppm PEG 1000) and rejection (~98%) performance over 45 h, indicating the great antifouling properties of the PES/SMA-PVA membrane. The surface-modified membrane exhibited a promising ability for CR rejection (92–98%), while showing poor ability for CaCl_2_ rejection (<10%). These results confirm that introducing a PVA layer onto the PES/SMA support led to the enhanced hydrophilicity, permeance, rejection performance, and antifouling properties of the NF membrane.

**Figure 12:**
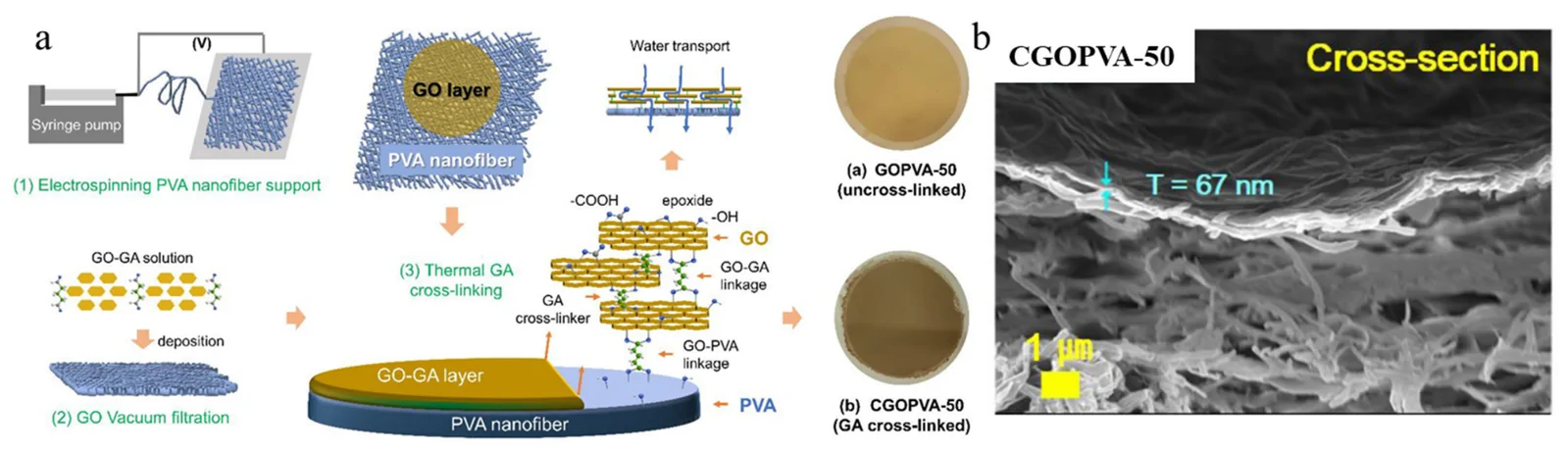
(**a**) Schematic diagram of membrane fabrication and (**b**) SEM micrograph of PVA/GO membranes (adapted from Park et al., 2021) [106].

In another study, Park et al. [106] developed PVA/GO NF membranes using a pressure-assisted self-assembly strategy. In that work, the porous PVA support was initially fabricated via electrospinning technique. In the next stage, GO nanosheets were chemically deposited on the surface of the membrane support using glutaraldehyde (crosslinker, GA) (Figure 12a). SEM analysis confirmed the deposition of GO nanosheets with a thickness of 67 nm on the PVA support (Figure 12b). The pure PVA membrane (i.e., CPVA) showed the lowest WCA value of ~10° after 10 s, while the physically and chemically modified membranes (GOPVA-50 and CGOPVA-50, respectively) showed higher values (~62.7° and 71.0°, respectively) owing to the relatively hydrophobic nature of GO nanosheets and the crosslinking reaction. The CGOPVA-50 membrane exhibited promising water permeance, ranging from 2.3 to 2.5 L·m^−2^·h^−1^·bar^−1^. Moreover, the CGOPVA-50 membrane exhibited a stable permeance (for Na_2_SO_4_ solution, ~2.4 L·m^−2^·h^−1^·bar^−1^) and a Na_2_SO_4_ rejection (95.8%) over time, indicating great antifouling properties of this modified membrane. These findings indicate that although the hydrophilicity of the pure PVA membrane reduced after surface modification, rejection performance and antifouling properties of modified membranes were significantly improved.

Chen et al. [107] developed PA-based NF membranes via grafting of positively charged monomers—choline chloride (CC), 2,3-dihydroxy-*N*,*N*,*N*-trimethylpropan-1-aminium chloride (DHTAC), and 2,3-epoxypropyltrimethylammonium chloride (EPTMAC)—onto the membrane surface through covalent linkages for drinking water purification. All modified membranes exhibited slightly higher water permeance (40.0–41.1 L·m^−2^·h^−1^·bar^−1^) compared to the pure membrane (38.0 L·m^−2^·h^−1^·bar^−1^) due to the presence of positively charged groups on the membrane surface, which enhanced electrostatic interactions and hydration effects. The prepared membranes exhibited higher rejection for sulfate-based salts (Na_2_SO_4_ and MgSO_4_, 93.0–98.1%) in comparison with chloride-based salts (CaCl_2_ and NaCl, 12.0–31.0%) owing to the larger hydrated ion size of sulfate salts. The antifouling test was carried out using 0.5 g·L^−1^ BSA solution. Modified membranes showed higher FRR values up to 99.2% compared with the unmodified PA membrane (83.6%), indicating the improved antifouling behavior of modified membranes due to the repulsion forces resulting from the presence of positively charged groups on the membrane surface. Such findings indicate that chemically modified membranes can offer stable transport and antifouling properties owing to the strong covalent bonds between the active layer and the membrane support.

#### 2.2.3. Layer-by-Layer Assembly

One of the common techniques to prepare polymeric NF membranes with excellent antifouling properties is the layer-by-layer (LbL) assembly method [108,111]. In this modification strategy, a thin selective separation layer is formed by alternately coating positively and negatively charged materials (typically polycations, polyanions, and NPs) onto the membrane surface through electrostatic interactions. The membrane is sequentially exposed to solutions (dipping into a positively charged material → rinsing → dipping into a negatively charged material → rinsing → drying) [108,109]. This modification can be carried out by dip coating, spin coating, and spray coating techniques. LbL membranes prepared by the mentioned methods can suffer from a leaching phenomenon, which leads to instability in membrane properties [109,110,111]. Therefore, to enhance chemical and mechanical stability, crosslinked LbL films can be prepared. Such membranes can offer high selectivity as well as antifouling properties, and owing to the low thickness of the layers, they can have excellent permeance. However, this method has some disadvantages [108,110]. First, this method is time-consuming due to multiple deposition cycles. Moreover, operating at high pressures may delaminate the membrane if it is not properly optimized. The stability issue is another demerit of these membranes, which can be mitigated by crosslinking [109,110].

**Figure 13:**
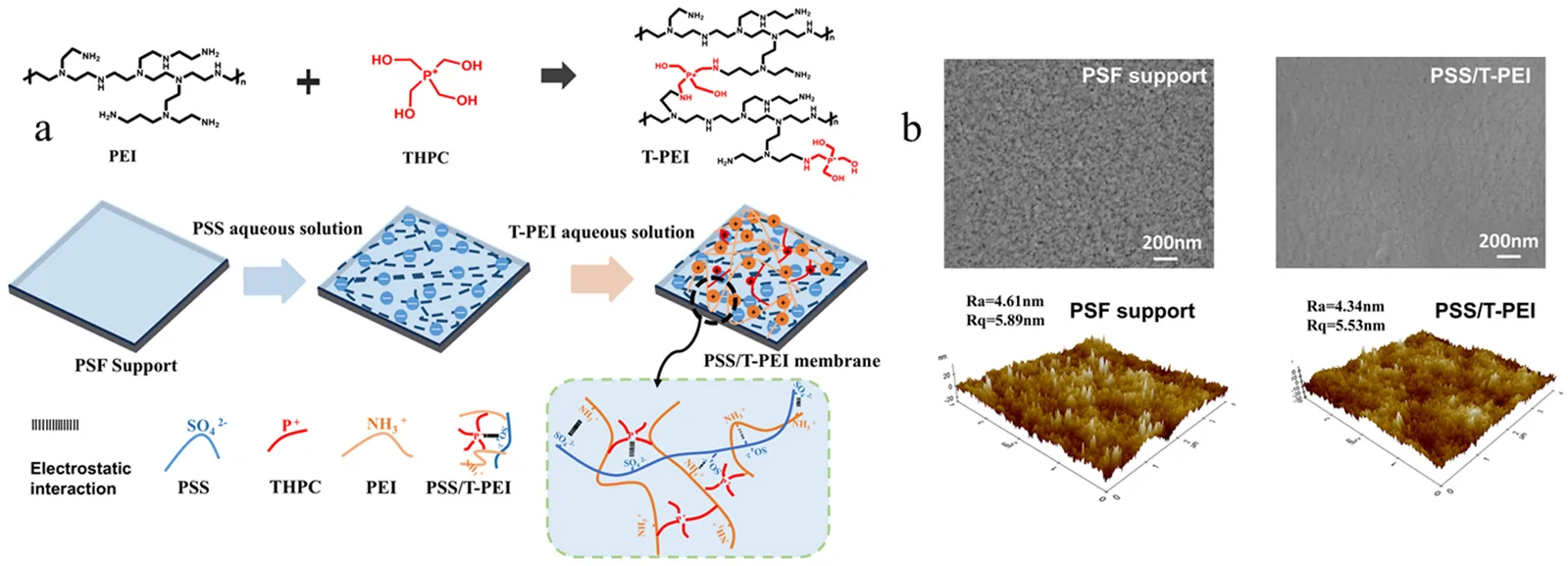
(**a**) Schematic diagram of membrane fabrication and (**b**) SEM and AFM images of developed PSF/PSS/THPC-PEI membranes (adapted from Jiang et al., 2026) [108].

Jiang et al. [108] developed high-performance LbL NF membranes for heavy metal removal. To prepare such membranes, the PSf support was initially immersed in polystyrenesulfonate (PSS, polyanion) possessing sulfonate groups (–SO_3_^−^). In the next stage, the coated support was immersed in a solution containing polyethyleneimine (PEI, polycation, 0.9 wt.%) and tetrakis(hydroxymethyl)phosphonium chloride (THPC, at 0.3, 0.4, 0.5, 0.6, and 0.7 wt.%) (Figure 13a). The SEM and atomic force microscopy (AFM) images showed a porous structure for the pristine PSf membrane. It can be observed that the PSf surface was effectively covered with PSS, THPC, and PEI, leading to decreased surface roughness of the LbL membrane compared to the pristine membrane (Figure 13b). The modified LbL membranes showed high water permeance (6.1 L·m^−2^·h^−1^·bar^−1^) and heavy metal removal performance (99.1%, 97.8%, and 97.6% for Cr^3+^, Ni^2+^, and Co^2+^, respectively) even in comparison with commercial membranes. Additionally, the antifouling test was performed over different cycles of water and lysozyme (LYZ, foulant) filtration. The modified membranes exhibited high FRR values (up to 94.7%), indicating their excellent antifouling properties. These findings confirm that NF membranes modified by the LbL assembly technique can exhibit promising transport and antifouling properties owing to the presence of positively and negatively charged compounds on the surface and within the thin active layer.

In another study, Dong et al. [109] designed hollow fiber LbL NF membranes for water desalination. To fabricate these membranes, PSS (as a polyanion) containing sulfonate groups (–SO_3_^−^) was initially coated on PES hallow fiber membranes. Then, polyallylamine (PAH, as a polycation) possessing amine groups (–NH_2_/–NH_3_^+^) was coated onto the PES/PSS membrane to prepare a PES/PSS/PAH membrane. The coating process was repeated 2.5 times to obtain a PES/PSS/PAH/PSS/PAH/PSS which is named (PSS/PAH)_2.5_. Subsequently, the (PSS/PAH)_2.5_ membrane was crosslinked using a GA solution for 10 min to obtain (PSS/PAH)_2.5-C_. Both LbL membranes exhibited relatively stable artificial seawater (ASW) permeance (~13.5 L·m^−2^·h^−1^·bar^−1^) and MgCl_2_ rejection (91–98%) over 300 h of filtration, indicating the promising antifouling properties of the membranes. The (PSS/PAH)_2.5-C_ membrane demonstrated slightly lower permeance and rejection values than the (PSS/PAH)_2.5_ membrane, due to the crosslinking reaction and decreased membrane hydrophilicity. Such observations indicate that LbL membranes can exhibit favorable transport and antifouling properties owing to the presence of charged polymers in their structure. However, harsh crosslinking conditions, such as high concentrations of crosslinker or long reaction times, can adversely influence LbL separation performance.

Dmitrenko et al. [110] studied how to enhance the NF separation performance of poly(2,6-dimethyl-1,4-phenylene oxide) (PPO) membranes in ethanol by employing bulk and surface modification techniques. First, GO was incorporated into the PPO matrix to improve the separation performance of membranes. Subsequently, the PPO/GO membranes were modified via the LbL assembly strategy to improve the transport properties, using polyethyleneimine (PEI, polycation) and polyacrylic acid (PAA, polyanion). It was found that increasing the concentration of GO (from 0 to 1.5 wt.%) led to a decrease in ethanol and dye solution permeance, while increasing dye rejection performance. The excessive addition of GO may result in a decrease in membrane hydrophilicity and GO agglomeration, which may, in turn, adversely reduce the permeability. The PPO/GO composite membrane containing 0.7 wt.% of GO was considered the optimal membrane (with desirable transport properties and rejection performance) to prepare LbL membranes. Furthermore, the influence of the number of PEI/PAA bilayers on the permeability and rejection performance of PPO/GO_0.7_ was investigated. The results revealed that the PPO/GO_0.7_/PEI/PAA_3bilayers_ membrane exhibited promising ethanol and dye permeability, as well as dye solution rejection performance (58.0–63.0%). These findings show that the excessive incorporation of GO and excessive increase in the number of modified layers can lead to a decline in membrane permeability. Therefore, optimization studies should be carried out to achieve a membrane with optimal transport properties.

In another research work, Baig et al. [111] designed high-performance LbL NF membranes for the desalination process. To develop such membranes, a commercial PA membrane was initially immersed in PAH (polycation) possessing ammonium groups (–NH_3_^+^). Subsequently, the PA/PAH membrane was immersed in a solution containing PAA (polyanion) comprising carboxylate groups (–COO^−^). Four membranes with different numbers of layers (0 (M-0), 10 (M-10), 20 (M-20), and 30 (M-30) layers) were prepared. The pristine PA membrane showed higher water permeance (~10.3 L·m^−2^·h^−1^·bar^−1^) compared to the PA/PAH/PAA membranes (~5.2–6.7 L·m^−2^·h^−1^·bar^−1^), due to the increased resistance to mass transfer caused by the deposited layers. Moreover, all membranes showed promising salt rejection performance (>98%). These observations indicate that the LbL assembly technique can significantly improve the antifouling properties, but membrane permeance may decrease due to the increased resistance to mass transfer resulting from the deposited layers.

#### 2.2.4. Plasma Treatment

One of the most useful techniques to tailor the surface of polymeric NF membranes without changing the bulk structure is plasma treatment method [112,113,114]. In this strategy, the membrane surface is exposed to ionized gases, including oxygen, nitrogen, argon, and air in order to introduce functional groups, such as –OH, –COOH, and –NH_2_ [113,114]. The main goal of this method is to improve hydrophilicity, surface charge, transport, and antifouling properties of polymeric NF membranes [112]. Moreover, this technique is environmentally friendly and adjustable. However, over-treatment can damage the active layer of the membrane, which leads to increase in pore size, causing poor selectivity [114]. Furthermore, reverting the membrane surface to its original state over time is another disadvantage of this method. For these reasons, plasma treatment method is less common than other techniques [113].

Dehghanpour et al. [112] developed high-performance plasma-treated NF membranes for the desalination process. To design such a membrane, a commercial polyethylene (PE) film was exposed to oxygen plasma (operating condition: 50 Watt, 0.09 kPa, and 10 s). In the next stage, a thin layer of PA was formed on the membrane surface through interfacial polymerization strategy. The plasma-treated membrane demonstrated lower WCA (~55.0°) compared with untreated membrane (~80.6°), thanks to generation of hydrophilic groups (–OH). However, the plasma treatment led to increased surface roughness of PE membrane from etching the surface. The untreated membrane exhibited higher water permeance (~15.3 L·m^−2^·h^−1^·bar^−1^) than plasma-treated membrane (~4.1 L·m^−2^·h^−1^·bar^−1^), due to the thin and undesirable formation of PA layer on the surface of hydrophobic PE membrane. Nonetheless, plasma-treated membrane showed much higher salt rejection values than untreated one, owing to the successful formation of the PA layer on the membrane surface, resulting from improved hydrophilicity during plasma treatment. Additionally, modified membrane showed higher FRR value (~53%) than unmodified membrane (~38%), confirming improved antifouling properties of plasma-treated membrane owing to the presence of polar groups on its surface. These finding indicate that plasma treatment can significantly improve hydrophilicity, rejection performance, and antifouling properties of polymeric NF membranes.

Kiamehr et al. [113] enhanced the separation performance of commercial PA NF membranes using an Air–Ar plasma treatment (operating condition: 25 W, 50 mtorr, and 120 s). Five plasma-treated membranes with different gas ratios (air–Ar, *v*/*v*: 0–100% (M_1_), 100–0% (M_2_), 80–20% (M_3_), 20–80% (M_4_), 50–50% (M_5_)) were prepared to compare their properties with the unmodified PA membrane (M_0_). Fourier transform infrared spectroscopy (FTIR) analysis revealed the appearance of –OH and –NH_2_ bands in the plasma-treated membranes, compared with the M_0_ membrane, indicating successful surface modification. It was also found that the WCA of the M_0_ membrane was significantly reduced after the plasma treatment from 80.4° to 6.5–13.5°, confirming a considerable improvement in the surface hydrophilicity of the modified membranes. M_5_ membrane exhibited the highest water permeance of 1.7 L·m^−2^·h^−1^·bar^−1^, which was much higher than that of the unmodified membrane (0.1 L·m^−2^·h^−1^·bar^−1^). However, the M_0_ membrane showed higher salt rejection (90.0%) than the M_5_ membrane (76.4%). These results confirm that plasma treatment can significantly enhance membrane morphology and hydrophilicity, leading to improved transport properties. However, there is a trade-off between permeance and rejection in these membranes, which requires careful membrane optimization.

In another study, Kiamehr [114] developed high-performance plasma-treated NF membranes for desalination process. For this purpose, a PVC membrane was initially fabricated using the NIPS method. Subsequently, the surface of the dried PVC membrane was exposed to air plasma at different discharge powers (0, 20, 40, and 60 W) for 5 min. The FTIR spectra revealed the presence of amine and amide functional groups in plasma-treated membranes in comparison with the pristine PVC membrane. The plasma-treated membranes exhibited high water permeance and Na_2_SO_4_ rejection performance (1.3–5.2 L·m^−2^·h^−1^·bar^−1^ and 58–85%, respectively). These findings indicate that the separation performance of polymeric NF membranes can be considerably enhanced by plasma treatment. However, overexposure can damage the membrane surface, and membrane properties may revert to its original state due to the lack of strong interactions.

#### 2.2.5. Interfacial Polymerization

Another promising approach to designing polymeric NF membranes with superior properties is the interfacial polymerization (IP) technique [115,117,118]. In this method, a chemical reaction occurs between two monomers distributed in immiscible phases (i.e., aqueous and organic). This chemical process merely takes place at the interface, where an ultrathin and highly dense polymer layer forms [115,116]. For polymeric NF membranes, this ultrathin layer (10–100 nm) is developed on top of a porous support, and it provides high water permeance and rejection [118]. Furthermore, the porous support acts as a backbone and ensures the mechanical stability of the membrane. The IP technique is extensively used owing to its advantages, such as its speed, scalability, and tunability through key factors, including monomer concentration, reaction time, and additives [116]. However, this method has some limitations, such as poor control over film uniformity and a trade-off between permeance and selectivity [117,118].

**Figure 14:**
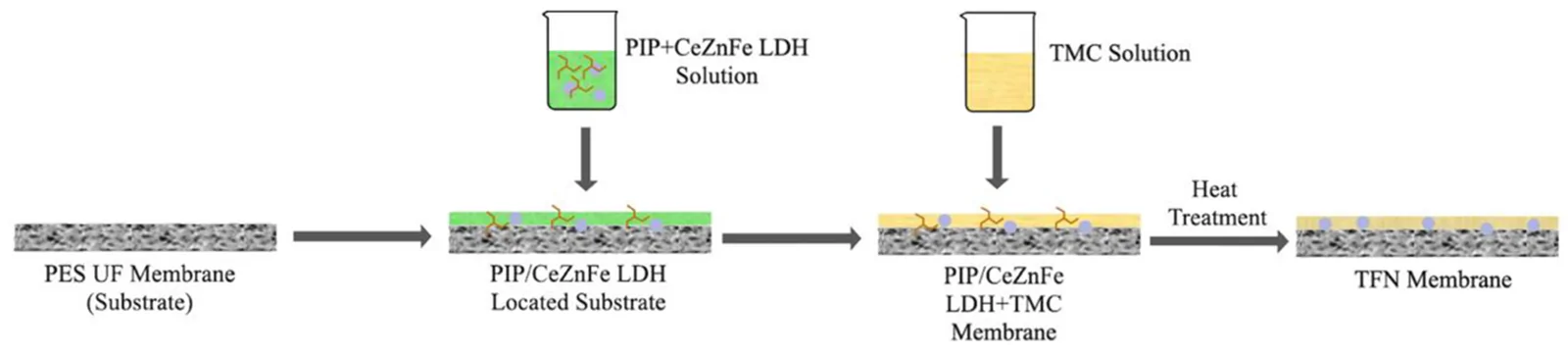
Schematic diagram of the fabrication of PES/PA-CeZnFe LDH membranes (adapted from Balcik et al., 2023) [115].

Balcik et al. [115] developed TFC membranes using the IP technique. For this purpose, a thin layer of PA was developed onto the surface of a commercial UF PES membrane by IP between piperazine (PIP, aqueous phase) and 1, 3, 5-benzene tricarboxylic chloride (TMC, organic phase). Four membranes containing different content of CeZnFe-layered double hydroxide (LDH) NPs in the PIP solution (0 (M_0_), 0.025 (M_1_), 0.05 (M_2_), and 0.1 (M_3_) wt.%) were prepared (Figure 14). The PES/PA-CeZnFe LDH membrane exhibited higher water flux than PES/PA membrane owing to the enhanced hydrophilicity of these membranes. However, the reference membrane demonstrated higher NaCl, Na_2_SO_4_, CR, and reactive red (RR) rejection values than modified membranes. These observations may be explained by morphological changes (i.e., increasing pore size) resulting from addition of CeZnFe LDH NPs. Such findings indicate a clear trade-off between membrane flux and (salt/dye) rejection, where further investigations are needed to obtain optimal NF membranes.

**Figure 15:**
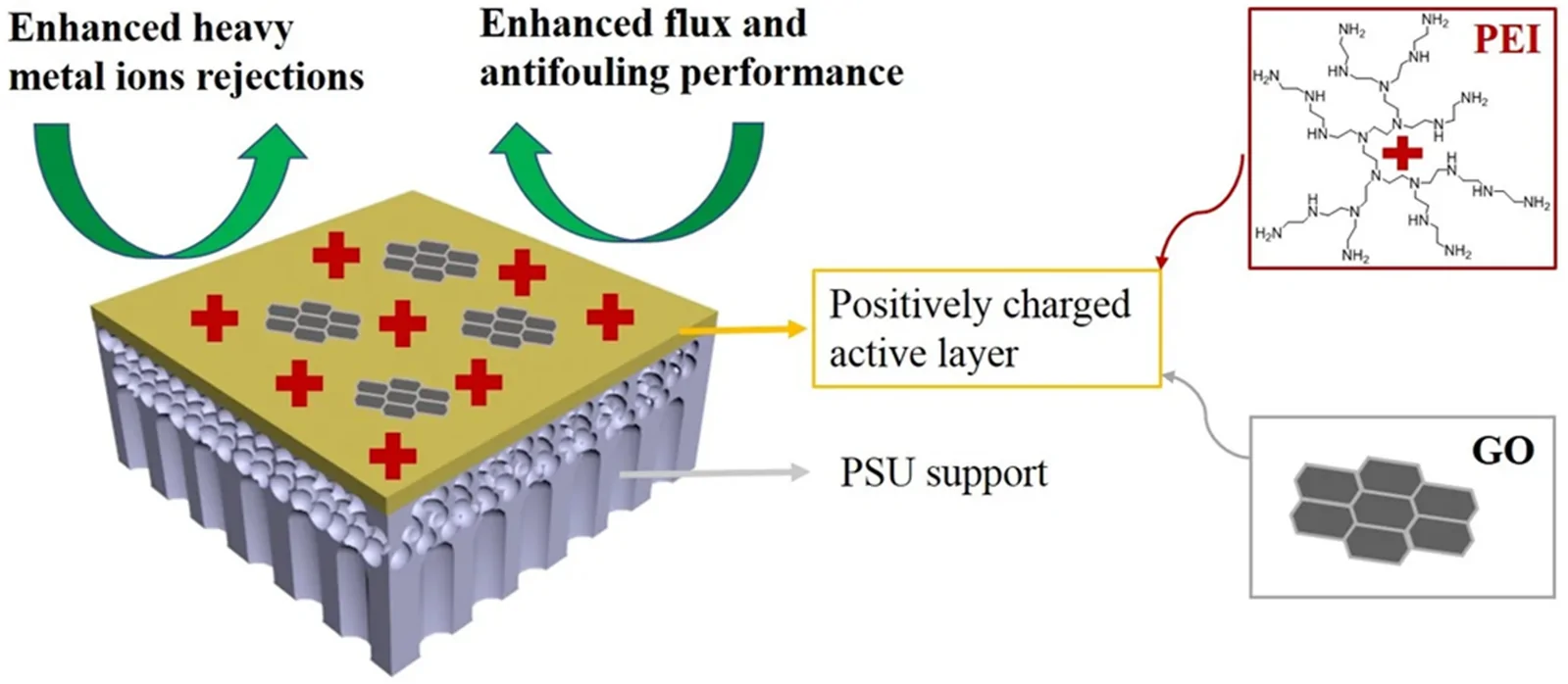
Schematic illustration of the modified PSf/PEI-GO membranes (adapted from Shao et al., 2020) [116].

In another study, Shao et al. [116] developed positively charged thin film nanocomposite (TFN) membranes via IP, forming a layer of PA on the surface of a UF PSf membrane (Figure 15). This was achieved through a reaction between PEI and TMC. Five membranes were fabricated by incorporating GO nanosheets at different concentrations in the aqueous solution (0 (TFC-PEI-blank), 10 (TFN-PEI-GO-10), 20 (TFN-PEI-GO-20), 30 (TFN-PEI-GO-30), and 40 (TFN-PEI-GO-40) ppm). All membranes containing GO nanosheets showed higher water permeance (~11.6 to 14.1 L·m^−2^·h^−1^·bar^−1^) than the TFC-PEI-blank membrane (~10.3 L·m^−2^·h^−1^·bar^−1^) owing to their improved hydrophilic properties. The salt rejection values showed that the addition of GO nanosheets did not significantly increase the salt rejection performance, indicating that the addition of GO at low concentrations could not enhance this property. Both TFC and TFN membranes demonstrated excellent potential for heavy metal removal (>91%). These findings indicate that the addition of GO nanosheets, even at low concentrations, could improve hydrophilicity and water permeance of PSf-based membranes owing to the presence of polar functional groups (e.g., –OH and –COOH).

Adeniyi et al. [117] designed TFC membranes using the IP of PIP (aqueous phase) and TMC (organic phase), where cellulose nanocrystals (CNC) were added to the aqueous solution. To evaluate the influence of CNC addition on the membrane properties, two membranes were fabricated—PT (without CNC) and PTUC (containing 0.21 g of CNC). The WCA measurements exhibited that the hydrophilicity of the PTUC membrane improved (~71°) in comparison with the PT (~81°) owing to the excellent hydrophilic properties of CNC possessing a number of polar hydroxyl groups (e.g., –OH). Moreover, the PTUC membrane showed higher pure water flux and nitrate rejection than the unmodified membrane. Such results can be explained by improved surface charge and hydrophilicity resulting from CNC incorporation.

**Figure 16:**
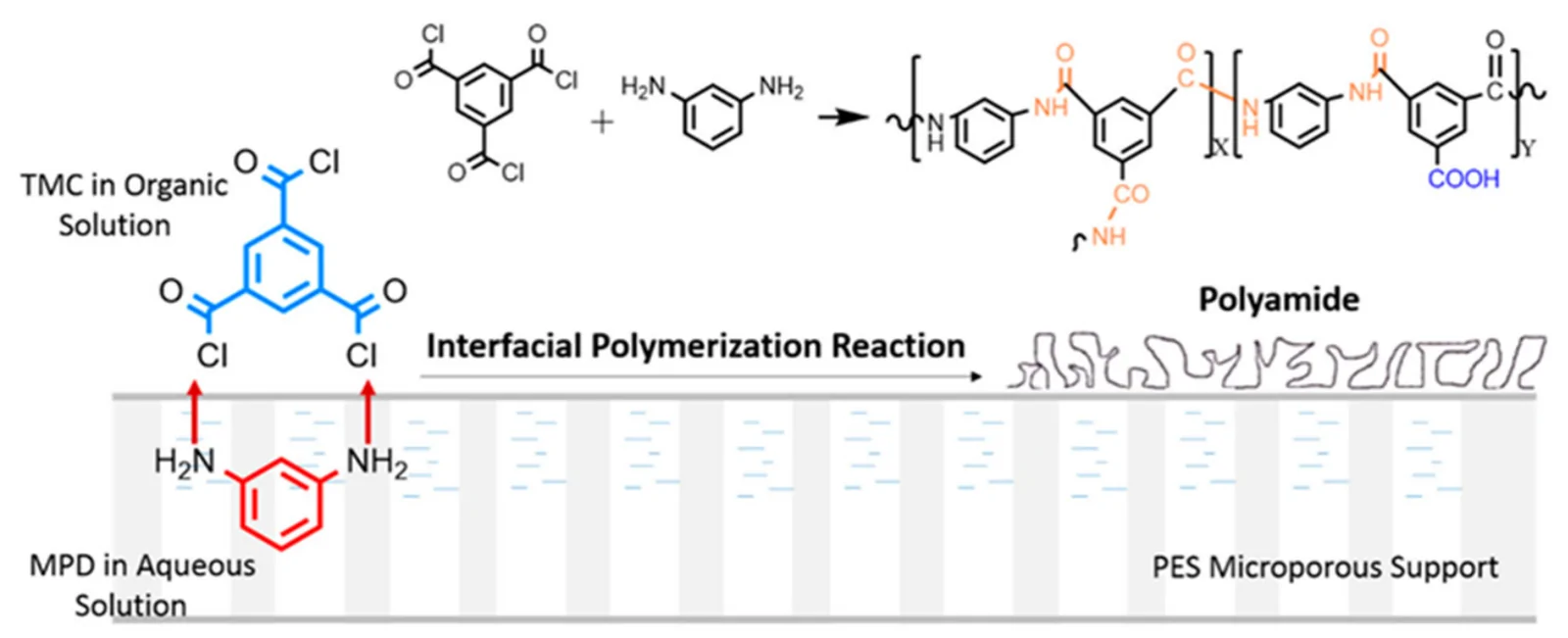
Schematic illustration of IP reaction between MPD and TMC at the PES porous support (adapted from Wang et al., 2022) [118].

In another study, Wang et al. [118] developed a series of NF membranes by coating various amounts (0, 1, 3, 5, 7, and 9 mL) of double-walled carbon nanotubes (DWCNTs) onto a PES support, followed by forming a PA active layer on top through the IP of m-phenylenediamine (MPD) and TMC (Figure 16). Additionally, the WCA of PES/PA-DWCNT membranes reduced with higher DWCANTs loading owing to the presence of functional groups (e.g., –OH, –COOH, and –C=O) and changes in surface roughness. Such functional groups enhanced the membrane surface hydrophilicity since they can strongly interact with water molecules. Moreover, incorporating DWCNTs led to an increase in water permeance (up to 21.8 L·m^−2^·h^−1^·bar^−1^) owing to the improved surface hydrophilicity, enhanced specific surface area, and the formation of nanochannels. Nonetheless, salt rejection decreased with increasing amount of DWCNTs. Indeed, the addition of DWCNTs (particularly at high dosages) resulted in making the membrane less dense and crosslinked. This phenomenon created looser pathways in the membrane structure (active layer) leading to increased water permeance but decreased salt rejection. The findings reveal that the hydrophilic and transport properties of polymeric NF membranes can be significantly improved by IP. However, there is a trade-off between permeance and rejection, which requires further membrane optimization to obtain modified membranes with overall excellent properties. Table 4 presents an overview of the various surface modification techniques used for polymeric NF membranes.

## 3. Conclusions and Prospects

Today, polymeric NF membranes have become widely utilized in various separation processes—such as water treatment, food and beverage processing, pharmaceutical production, industrial processes, and agriculture—due to their promising selectivity, permeance, and affordability. However, these membranes are usually vulnerable to fouling due to the insufficient hydrophilicity of commonly used polymers, which adversely affects membrane separation performance and raises economic concerns. The present review article provides comprehensive insights into different modification techniques—supported by recent research findings—used to enhance the performance of polymeric NF membranes.

This review reveals that both bulk modification methods (like polymer blending and nanoparticle incorporation) and surface modification strategies (like physical surface coating, plasma treatment, chemical attachment, layer-by-layer assembly, and interfacial polymerization) possess distinct benefits and drawbacks. For example, although bulk modification methods have unique benefits, including operational simplicity, affordability, scalability, reversibility, and low environmental impact, such modified NF membranes suffer from a lack of stable hydrophilicity, permeation performance, and antifouling properties as the interactions in the bulk are usually physical, causing leaching of additives from the membrane matrix over time. Similar concerns apply to surface modification methods. Even though these modifications can significantly improve NF membrane separation performance, their main drawback is the non-uniform property distribution, referring to distinct properties in different regions of the membrane. This raises an important question: which modification method offers the most effective improvements for polymeric NF membranes?

A hybrid modification technique may have the potential to develop NF membranes with optimized hydrophilicity, morphology, transport properties, and antifouling performance. By combining different approaches, the individual drawbacks of each modification method may be overcome by the strengths of another technique. For instance, surface coating is a promising method to improve hydrophilicity, water flux, and rejection efficiency. However, the main disadvantage of this modification technique is the gradient of properties, which negatively influence membrane performance. This downside can be tackled by taking advantage of a bulk modification method (e.g., polymer blending or NP incorporation) to obtain a NF membrane with uniform morphological and transport properties. As a result, this idea warrants increased focus and exploration in future studies.

Another promising research direction to obtain a high-performance polymeric NF membrane is to integrate AI and machine learning (ML) into NF technology. This rapidly developing scientific area can be used for the analysis of huge experimental datasets to find links between membrane composition, preparation variables, and key performance metrics, which might not be possible through traditional experimental approaches alone. For instance, ML algorithms can optimize polymer blend ratios, NP loading, and operating conditions to improve permeance and antifouling properties. Furthermore, AI-driven models can assist in estimating membrane lifespan and fouling behavior. Therefore, this emerging technology can minimize experimental trial-and-error, thereby saving time and energy.

## Figures and Tables

**Table 1:** Overview of pressure-driven membrane processes showing the trade-off between particle removal efficiency, operating pressure, energy demand, and permeate flux.

Membrane	Pore Size (μm)	Removes Well	Pressure (Bar)	Energy Demand	Flux
MF	~0.1–1.0	Suspended solids, algae, bacteria	0.1–2	Very low	Very high
UF	~0.01–0.1	Bacteria, protozoa, colloids	1–5	Low	High
NF	~0.001–0.01	Viruses, small organics, hardness	5–40	Medium	Medium
RO	Dense	Nearly all particles and salts	10–70	High	Low

**Table 2:** A summary of review papers focused on NF membranes.

Main Theme	Year	Ref.
Reviews improved NF membranes incorporating nanoparticles for effective saline water treatment	2020	[84]
Reviews recent advances in preparation and performance of organic solvent NF membranes	2021	[85]
Reviews design, preparation, and applications of loose NF membranes for dye and salt separation	2022	[31]
Reviews NF membrane types and their applications in water treatment	2023	[25]
Reviews fabrication methods and applications of NF membranes for heavy metal removal from aqueous solutions	2023	[86]
Reviews factors affecting NF membrane fouling and advances in fouling prediction using ML	2024	[39]
Reviews targeted preparation and optimization techniques for positively charged NF membranes in advanced water treatment applications	2025	[87]
Reviews materials, mechanisms, and applications of alkali-resistant NF membranes for harsh industrial conditions	2025	[88]
Reviews NF membrane efficiency and mechanisms for removing poly-fluoroalkyl substances from water and wastewater	2026	[89]
Reviews materials, mechanisms, and efficiency of NF membranes for Li^+^/Mg^2+^ separation	2026	[90]

**Table 3:** Summary of NF membranes modified by bulk modification techniques.

Modification Method	Membrane Composition	Testing Conditions	Highest Pure Water Permeance (L·m^–2^·h^–1^·bar^–1^)	Rejection Rate (%)	Ref.
Polymer Blending	Cellulose/Chitosan	Pressure: 10–30 bar Solute concentration: – Cell surface area: 53.0 cm^2^	95.3	Oil: 44.0–98.6	[73]
	PES/sPSf	Pressure: 3 bar Solute concentration: 0.2 ppm Cell surface area: 10.0 cm^2^	35.5	PEG: 58.0–98.0 LVFX: 96.0–99.0 MgCl_2_: <5.0	[69]
	PPEES/PAN/SBA-15	Pressure: 5–8 bar Solute concentration: 100 ppm Cell surface area: 28.0 cm^2^	13.3	Polyphenols: 65.9–97.8 Flavonoids: 10.8–50.3 MgSO_4_: 15.1–56.2	[70]
	PPSU/PES	Pressure: 9–10 bar Solute concentration: 20 ppm Cell surface area: 50.0 cm^2^	37.8	RB: 62.0–91.0	[71]
	PES/sPVC	Pressure: 5 bar Solute concentration: 1000 ppm Cell surface area: 11.9 cm^2^	34.1	Na_2_SO_4_: 53.0–67.0	[72]
	PPSU/PES	Pressure: 3 bar Solute concentration: – Cell surface area: 16.0 cm^2^	13.4	4-NP: 81.0–98.0	[91]
	PES/PES–OH	Pressure: 1 bar Solute concentration: – Cell surface area: –	200.2	DB38: >99.0 NaCl: <5.0	[92]
	PSf/PES–COOH	Pressure: 1 bar Solute concentration: 100–1000 ppm Cell surface area: –	230.0	CB: 62.0–97.0 NaCl: <5.0	[93]
Nanoparticle Incorporation	CA/PES/TiO_2_	Pressure: 6 bar Solute concentration: – Cell surface area: 19.6 cm^2^	14.9	NaCl: 63.5–76.8	[94]
	PEES/ZnO	Pressure: 13.8 bar Solute concentration: 1000 ppm Cell surface area: –	7.7	HA: 94.4–98 MgCl_2_: 90.0–98.8 NaCl: 80.3–92.3 MgSO_4_: 52.3–75.9 Na_2_SO_4_: 46.8–69.7	[95]
	PVDF/AGO	Pressure: 1–4 bar Solute concentration: – Cell surface area: 50.0 cm^2^	170.2	BSA: 88.2–98.2 MO:86.6–96.6 MB:78.3–88.5	[96]
	PES/Ag	Pressure: 9 bar Solute concentration: 1000 ppm (salts) and 30 ppm (dyes) Cell surface area: –	36.0	NaCl: 32.0–57.0 MgSO_4_: 26.0–67.0 CaCl_2_: 27.0–41.0 MB: 41.0–98.0 RB: 42.0–85.0 CR: 65.0–99.0	[97]
	PES/sPES/Ti_3_C_2_T_X_	Pressure: 1 bar Solute concentration: 1000 ppm (salts) and 10 ppm (dyes) Cell surface area: 8 cm^2^	24.0	NaCl: 4.0–9.0 Na_2_SO_4_: 7.0–15.0 MgSO_4_: 11.0–16.0 MB: 95.0–97.0 MO: 21.0–76.0 Methyl blue: 55.0–90.0 Metanil Y: 35.0–96.0 Saffron T: ~98.0	[98]
	PES/Fe_3_O_4_-PVP	Pressure: 5 bar Solute concentration: 1000 ppm Cell surface area: 11.9 cm^2^	2.0	Na_2_SO_4_: 82.0–90.0	[99]

**Table 4:** Summary of NF membranes modified by surface modification techniques.

Modification Method	Membrane Composition	Testing Conditions	Highest Pure Water Permeance (L·m^–2^·h^–1^·bar^–1^)	Rejection Rate (%)	Ref.
Physical Surface Coating	NF90/PDA	Pressure: 4–6 bar Solute concentration: 0.005 ppm Cell surface area: 137 cm^2^	9.8	NaCl: 90.8–93.2	[100]
	PSf/A-PDA-KMnO_4_-PEI	Pressure: 4 bar Solute concentration: 500 ppm (salts) and 100 ppm (dyes) Cell surface area: 13.7 cm^2^	82.7	NaCL: 47.0% MgCl_2_: 57.2% Na_2_SO_4_: 55.8% MgSO_4_: 68.2% CR: 99.7% MB: 88.5%	[101]
	PES/PVA	Pressure: 5–15 bar Solute concentration: 20–200 ppm Cell surface area: 19.6 cm^2^	8.0	Rhodamine: 93.0 NaCL: 40.0 MgSO_4_: 80.0 Na_2_SO_4_: 88.0	[102]
	PEIm/GO	Pressure: 1 bar Solute concentration: 10 ppm Cell surface area: –	179.0	RB: 80.0–91.0	[103]
Chemical Attachment	PES/Fe-TA-PEI	Pressure: 2 bar Solute concentration: 1000 ppm Cell surface area: 7.1 cm^2^	104.4	AB 8GX: 74.6–98.7 CR: 98.5 EBT: 99.5 BPB: 87.1 Na_2_SO_4_: 3.3 MgSO_4_: 7.1 MgCl_2_: 9.2 NaCl: 2.9–4.2	[104]
	PES/SMA-PVA	Pressure: 4 bar Solute concentration: 1000 ppm Cell surface area: 300 cm^2^	11.0 (for PEG solution)	PEG: ~98.0 CR: 92.0–98.0 CaCl_2_: <10.0	[105]
	PVA/GO	Pressure: 1–9 bar Solute concentration: 10 ppm Cell surface area: 4 cm^2^	2.5	MB: 76.9 Eosin Y: 98.1 Na_2_SO_4_: 95.8 MgSO_4_: 76.2 MgCl_2_: 27.1 NaCl: 49.6	[106]
	PA PA/CC PA/DHTAC PA/EPTMAC	Pressure: 4 bar Solute concentration: 1000 ppm Cell surface area: 22.1 cm^2^	41.1	Mg^2+^: 38.0–67.0 Ca^2+^: 39.0–65.0 Na^+^: 20.0–30.0 K^+^: 8.0–18.0 Cl^−^: 3.0–25.0 NO_3_^−^: 30.0–40.0 SO_4_^2−^: 64.0–94.0	[107]
Layer-by-Layer Assembly	PSf/PSS/THPC-PEI	Pressure: 6 bar Solute concentration: 100 ppm Cell surface area: –	6.1	Cr^3+^: 99.8 Ba^2+^: 97.4 Co^2+^: 96.1 Ni^2+^: 96.1 Mg^2+^: 94.5 Ca^2+^: 92.9 Cu^2+^: 92.5 Mn^2+^: 90.3 K^+^: 37.2 Na^+^: 34.2	[108]
	PES/PSS/PAH	Pressure: 3 bar Solute concentration: 500 ppm Cell surface area: 32 cm^2^	13.5	MgCl_2_: 91.0–98.0 MgSO_4_: 95.0–99.5 CaCl_2_: 96.0–99.5 SrCl_2_: 99.0–99.5 Na_2_SO_4_: 17.0–61.0 NaCl: 35.7–47.4 KCl: 59.0–65.0 H_3_BO_3_: <5.0	[109]
	PPO/GO PPO/GO/PEI/PAA	Pressure: – Solute concentration: 10 ppm Cell surface area: 20 cm^2^	~1.0 (for ethanol)	Sunset yellow: 58.0 CR: 63.0 Alphazurine FG: 58.0	[110]
	PA/PAH/PAA	Pressure: 10–30 bar Solute concentration: 1000 ppm Cell surface area: 20.6 cm^2^	10.3	MgSO_4_: >98.0 NaCl: >98.0	[111]
Plasma Treatment	PE/PA	Pressure: 15 bar Solute concentration: 2000 ppm Cell surface area: 36 cm^2^	15.3	NaCl: 35.0 MgCl_2_: 40.0 Na_2_SO_4_: 70.0 MgSO_4_: 50.0	[112]
	PA	Pressure: 6 bar Solute concentration: 1420 ppm Cell surface area: 11.9 cm^2^	1.7	Na_2_SO_4_: 76.6–92.5	[113]
	PVC	Pressure: 6 bar Solute concentration: 1420 ppm Cell surface area: 11.9 cm^2^	5.2	Na_2_SO_4_: 58.0–85.0	[114]
Interfacial Polymerization	PES/PA-CeZnFe LDH	Pressure: 1 bar Solute concentration: – Cell surface area: 14.6 cm^2^	78.0	Na_2_SO_4_: 40.0–57.0 NaCl: 2.0–12.0 CR: 92.0–98.0 RR: 74.0–91.0	[115]
	PSf/PEI-GO	Pressure: 5 bar Solute concentration: 2000 ppm Cell surface area: 70 cm^2^	14.1	Na_2_SO_4_: 91.0 MgSO_4_: 98.0 MgCl_2_: 99.0 NaCl: 44.0 CuCl_2_: 96.0 NiCl_2_: 95.0 Pb(NO_3_)_2_: 91.0 ZnCl_2_: 98.0 CdCl_2_: 96.0	[116]
	PSf/PA-CNC	Pressure: 5–10 bar Solute concentration: 60 ppm Cell surface area: 14.6 cm^2^	7.2	Nitrate: 91.0–94.0	[117]
	PES/PA-DWCNT	Pressure: 5 bar Solute concentration: 2000 ppm Cell surface area: 28.7 cm^2^	21.8	Na_2_SO_4_: 96.5–99.9 NaCl: 60.5–99.4	[118]

## Data Availability

No new data were created or analyzed in this study. Data sharing is not applicable.

## Abbreviations

1,3-PD	1,3-Propanediol
4-NP	4-Nitrophenol
AB 8GX	Alcian Blue 8GX
AFM	Atomic force microscopy
AI	Artificial intelligence
Al_2_O_3_	Aluminum oxide
A-PDA	Activated polydopamine
ASW	Artificial seawater
BDC	Butyl decarbonate
BPB	Bromop blue
BSA	Bovine serum albumin
CA	Cellulose acetate
CC	Choline chloride
CeO_2_	Cerium (IV) oxide
CNC	Cellulose nanocrystals
CR	Congo red
CS	Chitosan
DB38	Direct black
DHTAC	2,3-Dihydroxy-*N*,*N*,*N*-trimethylpropan-1-aminium chloride
DMAc	Dimethylacetamide
DWCNT	Double-walled carbon nanotubes
EBT	Eriochrome black T
EPTMAC	2,3-Epoxypropyltrimethylammonium chloride
Fe_2_O_3_	Iron (III) oxide (Fe_2_O_3_)
FRR	Flux recovery ratios
FTIR	Fourier transform infrared spectroscopy
GA	Glutaraldehyde
GO	Graphene oxide
HA	Humic acid
HMD	Hexamethylene diamine
IEP	Isoelectric point
IP	Interfacial polymerization
KMnO_4_	Potassium permanganate
LbL	Layer-by-layer
LDH	Layered double hydroxide
LVFX	Levofloxacin
LYZ	Lysozyme
MB	Methylene blue
MF	Microfiltration
MgCl_2_	Magnesium chloride
MHA	4-Morpholineacetate
ML	Machine learning
MMM	Mixed matrix membrane
MO	Methyl orange
MOF	Metal–organic framework
MPD	M-phenylenediamine
Na_2_SO_4_	Sodium sulfate
NF	Nanofiltration
NIPS	Non-solvent induced phase separation
NMH	*N*-(2-hydroxypropyl) morpholine
NP	Nanoparticle
NT	Nanotube
PA	Polyamide
PAA	Polyacrylicacid
PAH	Polyallylamine
PAN	Polyacrylonitrile
PEES	Poly(ether ether sulfone)
PEG	Polyethylene glycol
PEI	Polyethyleneimine
PEIm	Polyetherimide
PES	Polyethersulfone
PES-COOH	Carboxylated polyethersulfone
PI	Polyimide
PIP	Piperazine
PPEES	Poly(1,4-phenylene ether ether sulfone)
PPO	Poly(2,6-dimethyl-1,4-phenylene oxide)
PPSU	Poly(phenyl sulfone)
PSf	Polysulfone
PSS	Polystyrenesulfonate
PVA	Polyvinyl alcohol
PVC	Polyvinyl chloride
PVDF	Polyvinylidene fluoride
PVP	Polyvinylpyrrolidone
RB	Rose Bengal
RO	Reverse osmosis
RR	Reactive red
SBA-15	Santa Barbara Amorphous-15
SEM	Scanning electron microscopy
SiO_2_	Silicon dioxide
SMA	Poly(styrene-maleic anhydride)
TFC	Thin film composite
TFN	Thin film nanocomposite
THPC	Tetrakis(hydroxymethyl)phosphonium chloride
Ti_3_C_2_T_X_	Monolayer titanium carbide nanosheets
TiO_2_	Titanium dioxide
TMC	1, 3, 5-benzene tricarboxylic chloride
UF	Ultrafiltration
VIPS	Vapor-induced phase separation
WCA	Water contact angle
